# Supplementary material for: Multivariate extension of penalized regression on summary statistics to construct polygenic risk scores for correlated traits
Source: HGG Adv. 2023 May 20;4(3):100209. doi: 10.1016/j.xhgg.2023.100209 (PMC10276147; doi:10.1016/j.xhgg.2023.100209)
Supplement: Document S1. Figures S1–S9 and Tables S1–S4 [file mmc1.pdf]

HGGA, Volume *4*

Supplemental information

Multivariate extension of penalized regression on summary statistics to construct polygenic risk scores for correlated traits

Meriem Bahda, Jasmin Ricard, Simon L. Girard, Michel Maziade, Maripier Isabelle, and Alexandre Bureau

Supplemental information

1 Simulation parameters

For the mixture-of-four-genetic-covariance-matrices scenario, we generated the vector (β_{j1}, β_{j2}) of genetic effects for the SNP j for the trait 1 and trait 2 as follows :

$$\begin{pmatrix} \beta_{j1} \\ \beta_{j2} \end{pmatrix} \sim \begin{cases} N\left(\begin{bmatrix} 0 \\ 0 \end{bmatrix}, \begin{bmatrix} \sigma^2 & \sigma^2 r \\ \sigma^2 r & \sigma^2 \end{bmatrix}\right) & \text{with probability } \pi_1 \quad (C=1) \\ \begin{bmatrix} N\left(0, \sigma^2\right) \\ 0 \end{bmatrix} & \text{with probability } \pi_2 \quad (C=2) \\ \begin{bmatrix} 0 \\ N\left(0, \sigma^2\right) \end{bmatrix} & \text{with probability } \pi_3 \quad (C=3) \\ \begin{bmatrix} 0 \\ 0 \end{bmatrix} & \text{with probability } \pi_4 \quad (C=4) \end{cases} \tag{1}$$

with

$$\begin{aligned} &\cdot \operatorname{Cov}\left(\beta_{j1}, \beta_{j2}\right) = \operatorname{Cov}\left(\beta_{j1}, \beta_{j2} \mid C=1\right) \cdot P(C=1) = \sigma^2 r \pi_1, \\ &\cdot \operatorname{Var}\left(\beta_{j1}\right) = \operatorname{Var}\left(\beta_{j1} \mid C \leqslant 2\right) \cdot P(C \leqslant 2) = \sigma^2\left(\pi_1 + \pi_2\right), \\ &\cdot \operatorname{Var}\left(\beta_{j2}\right) = \operatorname{Var}\left(\beta_{j2} \mid C \in \{1,3\}\right) \cdot P(C \in \{1,3\}) = \sigma^2\left(\pi_1 + \pi_3\right), \\ &\cdot \pi_1 + \pi_2 + \pi_3 + \pi_4 = 1. \end{aligned} \tag{2}$$

We have 6 unknown parameters, $\pi_1, \pi_1, \pi_3, \pi_4, \sigma, r$, and 4 equations. We chose to specify the value of the parameters π_1 and σ in order to solve the equations.

It is important to note that we have the following constraints when we choose the value of π_1 :

$$\begin{aligned} \pi_1 &> \frac{\operatorname{Cov}\left(\beta_{j1}, \beta_{j2}\right)}{\sigma^2}, \\ \pi_1 &< min\left(\frac{\operatorname{Var}\left(\beta_{j1}\right)}{\sigma^2}, \frac{\operatorname{Var}\left(\beta_{j2}\right)}{\sigma^2}\right). \end{aligned}$$

Otherwise, we obtain negative values for the parameters π_2 and π_3, and a value of r greater than 1. We note that under the constant heritability and genetic covariance scenario, $\operatorname{Cov}\left(\beta_{j1}, \beta_{j2}\right) = \frac{1}{p}\rho_g$ and $\operatorname{Var}\left(\beta_{jk}\right) = \frac{1}{p}h_{gk}^2, k = 1, 2$, i.e. they are proportional to the prespecified heritabilities and genetic covariance.

The value of σ^2 was chosen according to the number of SNPs used in the simulations, in order to obtain targeted ranges of values for π_1. For the high polygenicity scenario, we set $\sigma^2 = 2.00e-6$ with 479,158 SNPs and $\sigma^2 = 2.26e-6$ with 423,552 SNPs in order to have a π_1 that varies within the interval [0.29 ; 0.45]. We then set $\pi_1 = 0.35$ and obtained $\pi_2 = 0.15$, $\pi_3 = 0.13$, $\pi_4 = 0.37$ and $r = 0.79$ by solving the system of equations (2). For the low polygenicity scenario, we set $\sigma^2 = 1.06e-5$ and $\pi_1 = 0.08$ and obtained $\pi_2 = 0.024$, $\pi_3 = 0.02$, $\pi_4 = 0.876$ and $r = 0.88$ by solving the system of equations (2). Table S1 presents the corresponding number of SNPs for each scenario.

For the scenario where the heritabilities and genetic covariance of the two traits depend on predictions from BLD-X model annotations we kept the same πs. Given the predicted contribution to heritability of either trait was negative for a number of SNPs and was thus set to 0 to begin with, the selection of causal SNPs for the simulation was restricted. We present below the steps for the simulations with 423,552 SNPs and high polygenicity. Similar steps were followed for 479,158 SNPs or low polygenicity.

1. The 148,243 SNPs causal for both traits (35% of 423,552) were selected among the 282,194 SNPs with $\hat{h}^2_{g1}(j) > 0$ and $\hat{h}^2_{g2}(j) > 0$.

2. The 59,445 SNPs causal for trait 1 only were selected among the SNPs remaining from step 1 and the 37,293 SNPs with $\hat{h}^2_{g1}(j) > 0$ and $\hat{h}^2_{g2}(j) \leq 0$.

3. The 50,607 SNPs causal for trait 2 only were selected among the SNPs remaining from step 1 not selected at step 2 and the 45,184 SNPs with $\hat{h}^2_{g1}(j) \leq 0$ and $\hat{h}^2_{g2}(j) > 0$.

4. The effect of all remaining SNPs was set to 0.

The non-zero SNP effects were then simulated according to equation (7) from the main text.

2 Genotyping quality control procedures

SNPs with the following quality problems were removed: missing call rate higher than 0.02, SNP mismatches with Haplotype reference consortium (HRC), SNPs which are indels, minor allele frequency difference with HRC > 0.2, palindromic SNP with frequency > 0.4, allele mismatch with HRC, duplicates, Hardy Weinberg equilibrium test p-value lower than 10^{-7}. From the first wave of SNP array genotyping using an Illumina Omni Express chips at Genome Québec, a total of 507 subjects were genotyped at 656,032 autosomal SNPs. A total of 33,848 SNPs were removed following quality control. Imputation using the Michigan Imputation Server resulted in a total of 39,127,678 imputed SNPs.

From the second wave of SNP array genotyping using an Illumina Global Screening Array at Genome Québec, a total of 615 subjects were genotyped at 691,719 autosomal SNPs. Two subjects were removed: one, thought to be a brother of three other subjects, turned out to be an half-brother after analysis with Prest-plus software and the other presented a sex disconcordance along with a high rate of Mendelian errors. A total of 188,854 SNPs were removed following quality control. Imputation using the Michigan Imputation Server resulted in a total of 39,131,578 imputed SNPs.

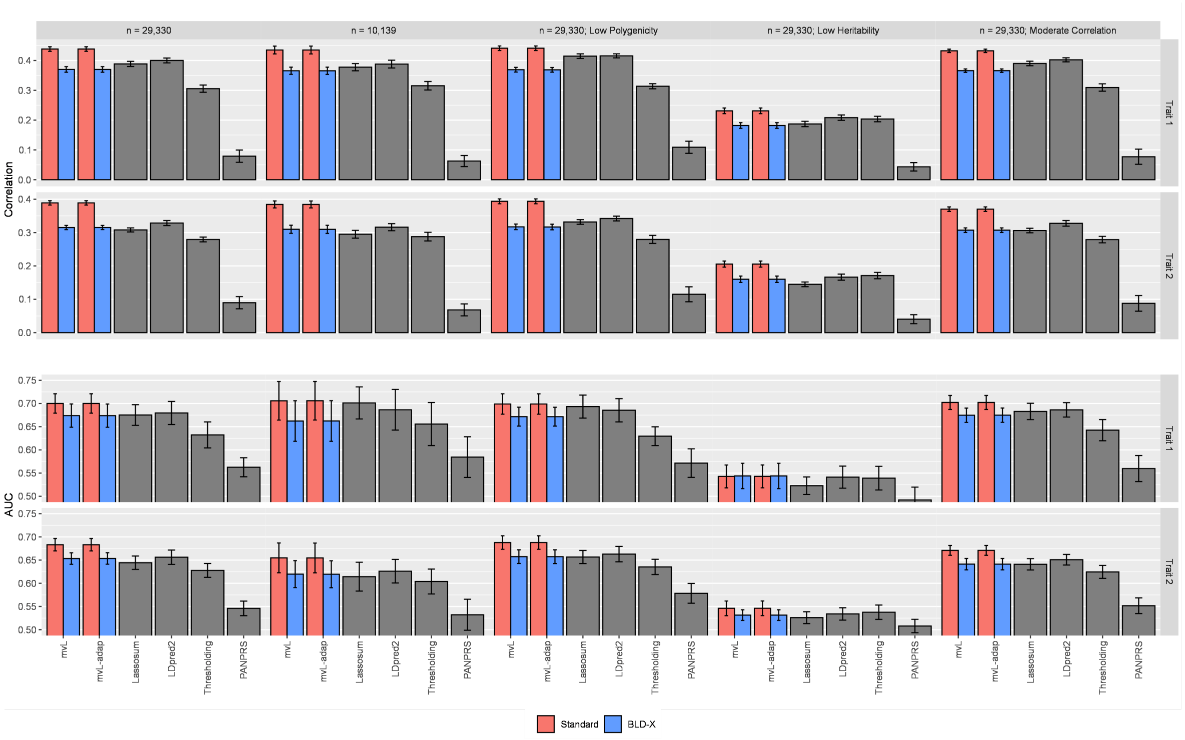

Figure S1: Comparison of PRS predictive performance for multiple variations of the simulation scenario with a mixture of four constant genetic covariance matrices. Top panel: Pearson correlation of the PRS with the true predictor. Bottom panel: area under the receiver operating curve (AUC) for the prediction of simulated traits by PRS. Mean and 95% confidence interval based on 20 replicates. The penalty parameter λ (for the penalized regression methods) and the threshold for thresholding were set to the values maximizing the correlation between the PRS and the trait y in a validation set. Models for heritability and covariance used in analysis : BLD-X: Baseline linkage disequilibrium model-cross trait; Standard: constant contribution of standardized genotypes of all SNPs.

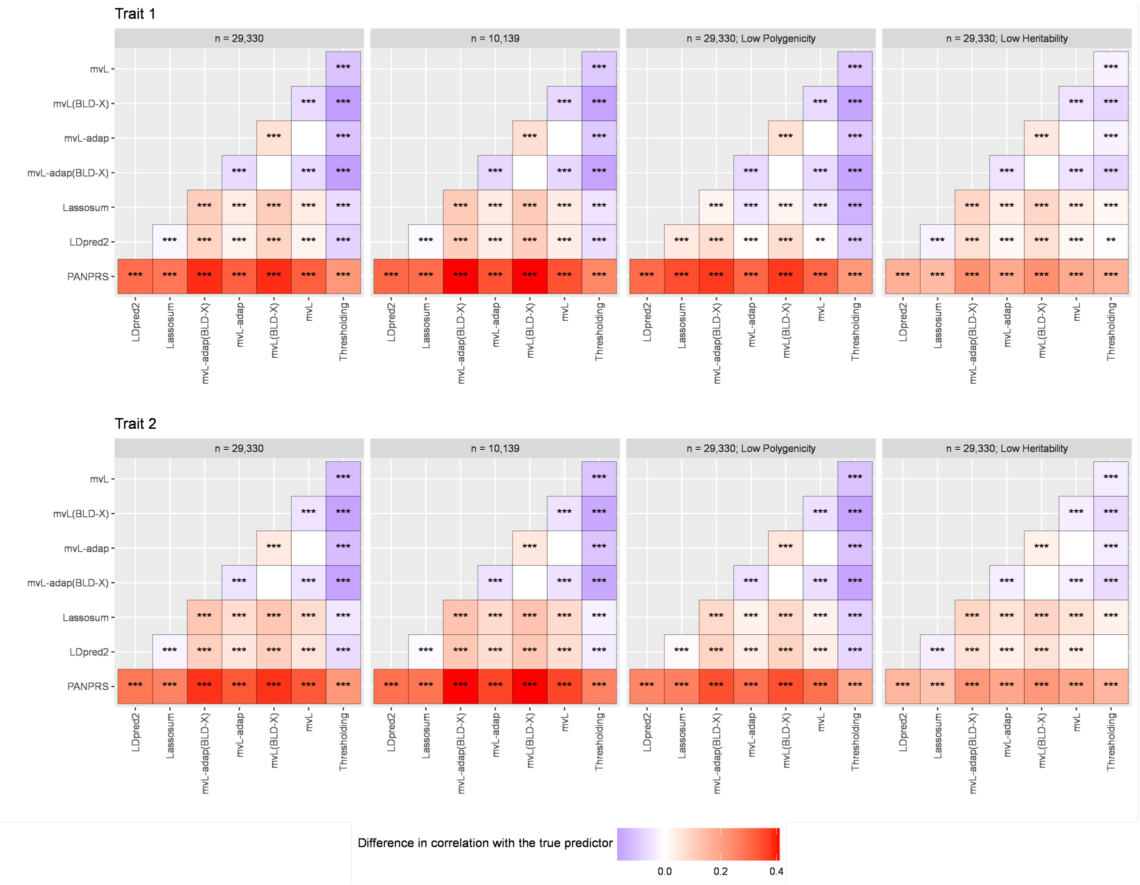

Figure S2: Difference in Pearson correlation of the PRS with the true predictor between methods under various simulation scenarios with heritability and genetic covariance of the two traits depending on genomic annotations. See legend of Figure 1 for the definitions of acronyms for the methods. Statistical significance of the difference evaluated by paired t-tests using 20 replicates, and p-values were corrected for multiple testing using the Bonferroni method. *: $p < 0.05$, **: $p < 5 \times 10^{-4}$, ***: $p < 5 \times 10^{-8}$

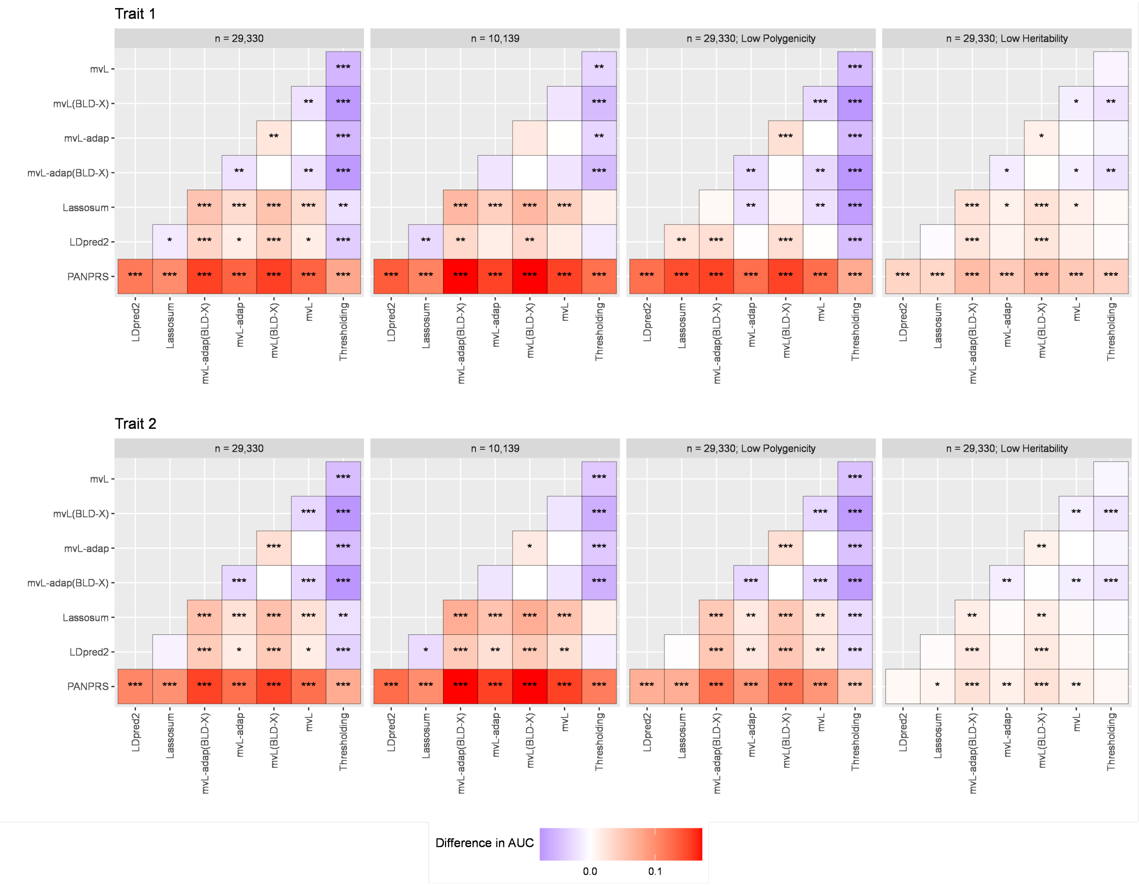

Figure S3: Difference in area under the receiver operating curve (AUC) for the prediction of simulated traits by PRS under various simulation scenarios with heritability and genetic covariance of the two traits depending on genomic annotations. See legend of Figure 1 for the definitions of acronyms for the methods. Statistical significance of the difference evaluated by paired t-tests using 20 replicates, and p-values were corrected for multiple testing using the Bonferroni method. *: $p < 0.05$, **: $p < 5 \times 10^{-4}$, ***: $p < 5 \times 10^{-8}$

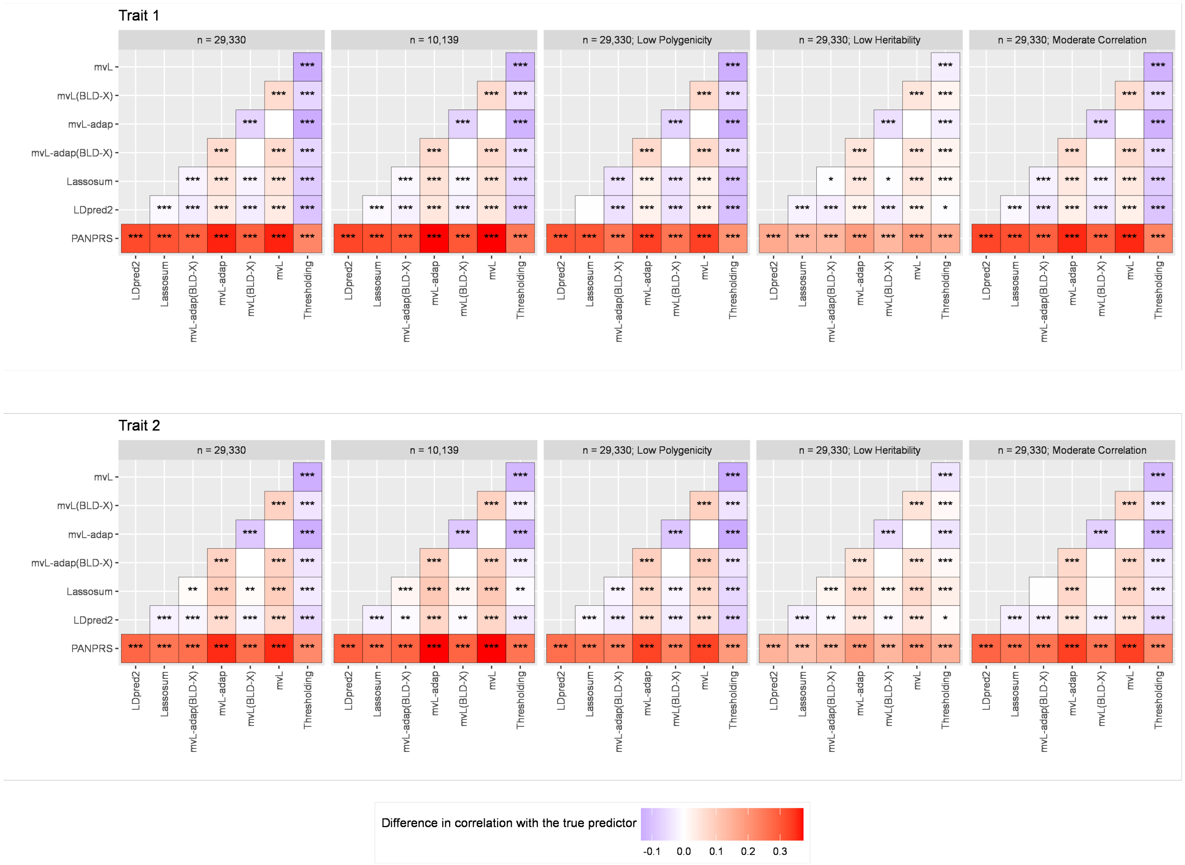

Figure S4: Difference in Pearson correlation of the PRS with the true predictor between methods under various simulation scenarios with a mixture of four constant genetic covariance matrices. See legend of Figure 1 for the definitions of acronyms for the methods. Statistical significance of the difference evaluated by paired t-tests using 20 replicates, and p-values were corrected for multiple testing using the Bonferroni method. *: $p < 0.05$, **: $p < 5 \times 10^{-4}$, ***: $p < 5 \times 10^{-8}$

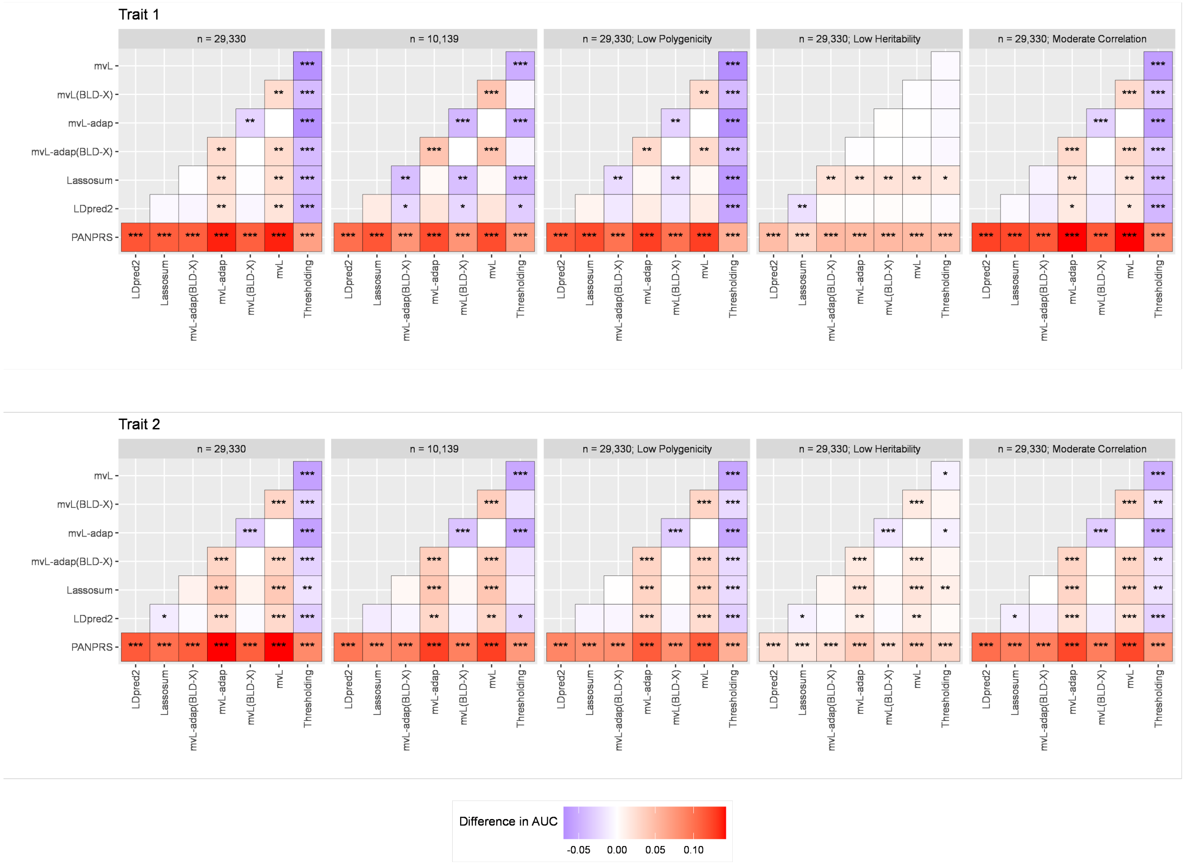

Figure S5: Difference in area under the receiver operating curve (AUC) for the prediction of simulated traits by PRS under various simulation scenarios with a mixture of four constant genetic covariance matrices. See legend of Figure 1 for the definitions of acronyms for the methods. Statistical significance of the difference evaluated by paired t-tests using 20 replicates, and p-values were corrected for multiple testing using the Bonferroni method. *: $p < 0.05$, **: $p < 5 \times 10^{-4}$, ***: $p < 5 \times 10^{-8}$

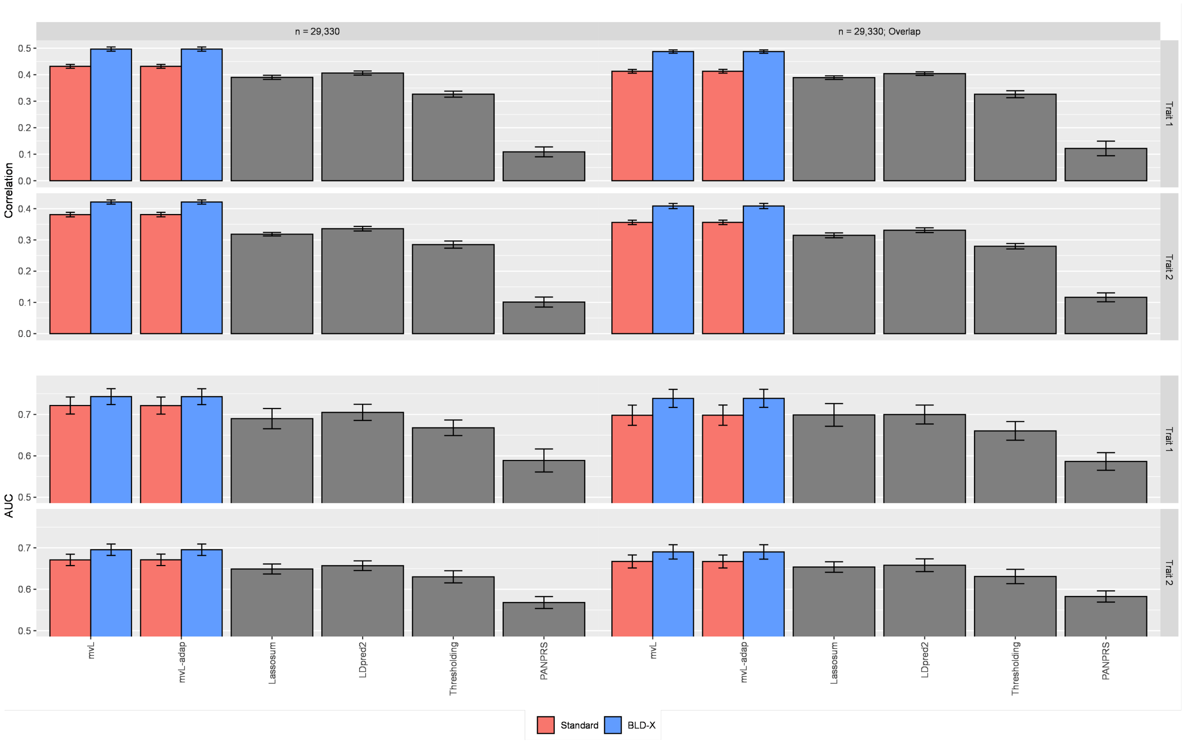

Figure S6: Comparison of PRS predictive performance in presence of simulated sample overlap with between-trait summary-statistics correlation $\hat{\rho}_o = 0.32$ compared to independent samples under the simulation scenario where heritability and genetic covariance of two traits depend on genomic annotations. Top panel: Pearson correlation of the PRS with the true predictor. Bottom panel: area under the receiver operating curve (AUC) for the prediction of simulated traits by PRS. Mean and 95% confidence interval based on 20 replicates. The penalty parameter λ (for the penalized regression methods) and the threshold for thresholding were set to the values maximizing the correlation between the PRS and the trait y in a validation set. Methods compared: mvL: Multivariate Lassosum with constant penalty, mvL-adapt: Multivariate Lassosum with adaptive penalty based on the initial estimates from mvL. Models for heritability and covariance used in analysis : BLD-X: Baseline linkage disequilibrium model-cross trait; Standard: constant contribution of standardized genotypes of all SNPs.

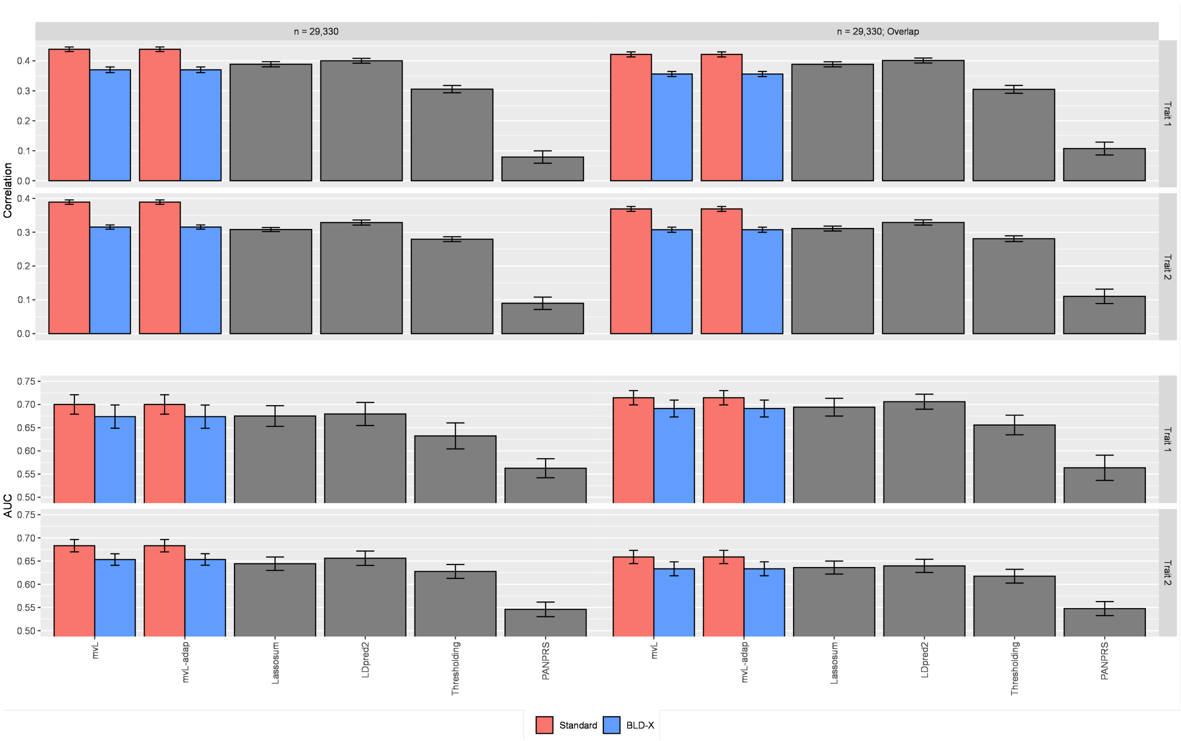

Figure S7: Comparison of PRS predictive performance in presence of simulated sample overlap with between-trait summary-statistics correlation $\hat{\rho}_o = 0.32$ compared to independent samples under the simulation scenario with a mixture of four constant genetic covariance matrices. Top panel: Pearson correlation of the PRS with the true predictor. Bottom panel: area under the receiver operating curve (AUC) for the prediction of simulated traits by PRS. Mean and 95% confidence interval based on 20 replicates. The penalty parameter λ (for the penalized regression methods) and the threshold for thresholding were set to the values maximizing the correlation between the PRS and the trait y in a validation set. Methods compared: mvL: Multivariate Lassosum with constant penalty, mvL-adapt: Multivariate Lassosum with adaptive penalty based on the initial estimates from mvL. Models for heritability and covariance used in analysis : BLD-X: Baseline linkage disequilibrium model-cross trait; Standard: constant contribution of standardized genotypes of all SNPs.

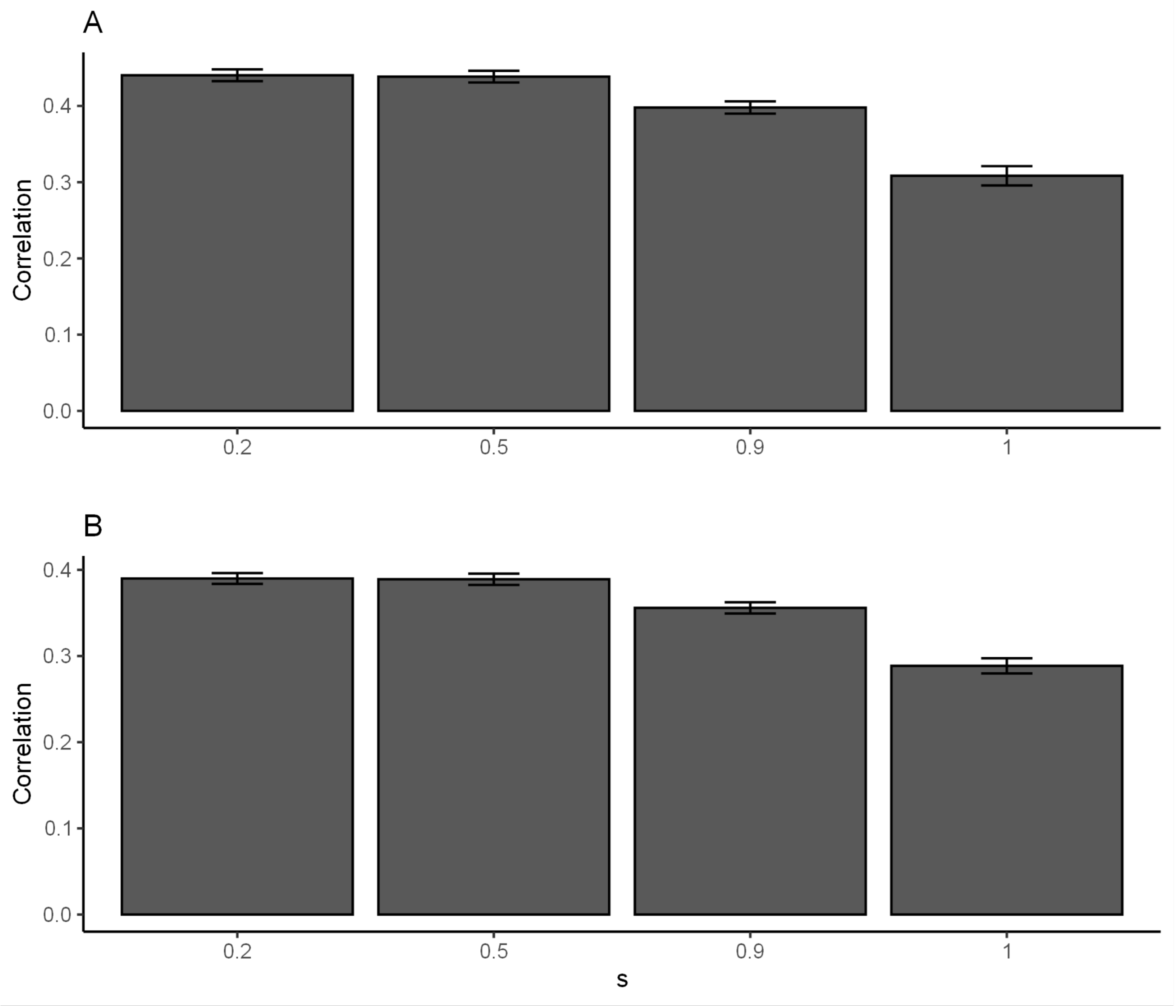

Figure S8: Pearson correlation of the PRS with the true predictor as a function of the s regularization parameter under the reference simulation scenario. A) Heritability and genetic covariance of two traits depending on genomic annotations B) Mixture of four constant genetic covariance matrices.

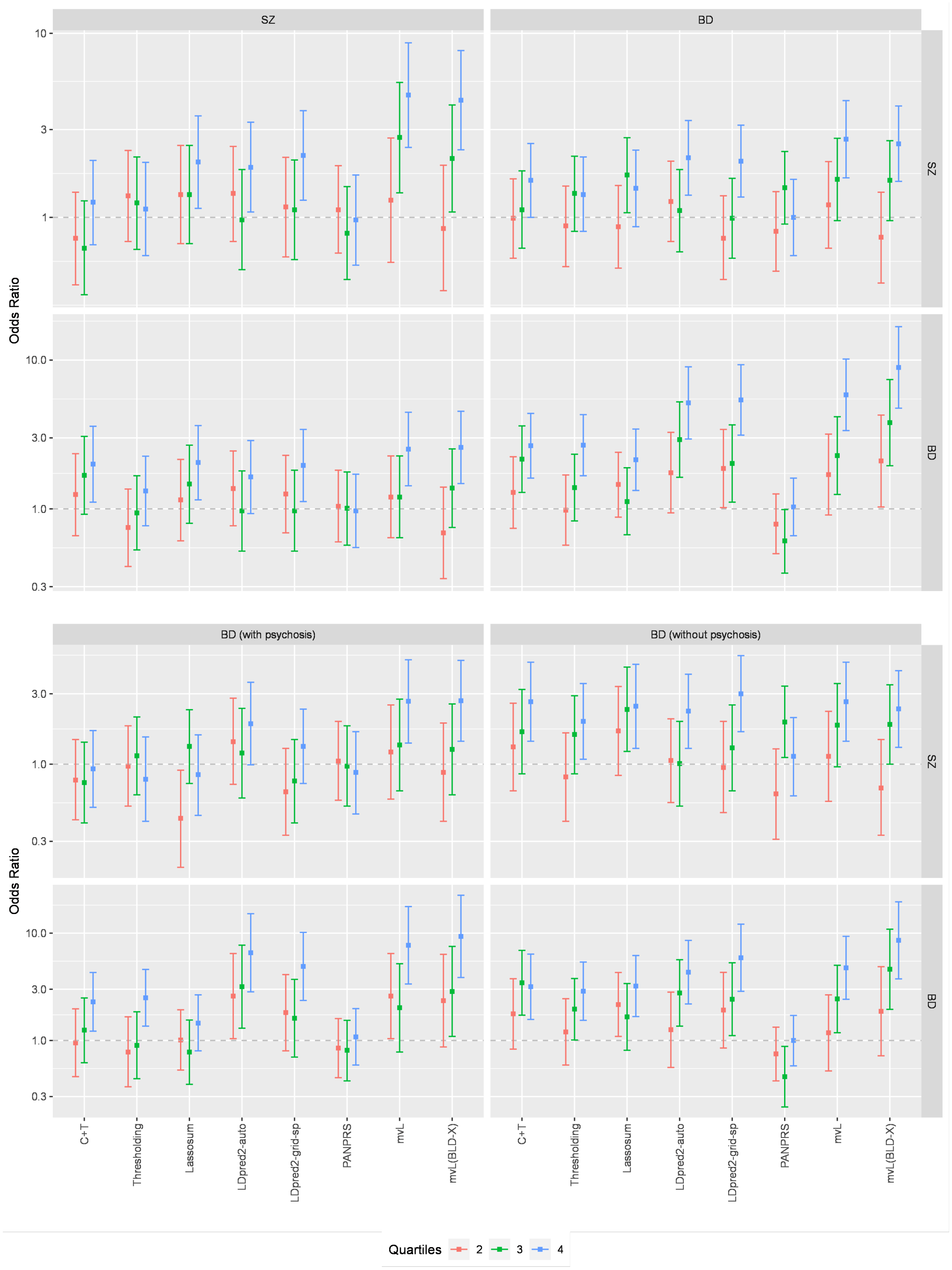

Figure S9: Odds ratios of psychiatric diagnoses for the second to fourth quartiles of PRS defined in the non-affected adult relatives compared to the first quartile based on various PRS definitions. Estimates and 95% confidence intervals. Each column is a predicted trait and each row a trait on which summary statistics were obtained to construct PRSs. SZ : schizophrenia, BD: Bipolar disorder. Schizoaffective disorder was excluded due to the small number of cases.

Table S1: Number of causal SNPs for each trait combination under different numbers of SNPs and polygenicity levels

Traits	π^a	High Polygenicity n = 10,139 p = 479,158	n = 29,330 p = 423,552	Low Polygenicity π^a	n = 29,330 p = 423,552
1 and 2	0.35	167,705	148,243	0.080	33,884
1	0.14	67,082	59,445	0.024	10,353
2	0.12	57,499	50,607	0.020	8,471
None	0.39	186,872	165,257	0.876	370,844

[a]: Proportion of causal SNPs in each case

Table S2: Features of the polygenic risk score methods

Method	Parameters	Use of validation sample	Heritability model	Clumping (in real data)
C + T[a]	P-value threshold, Correlation threshold	Yes	NA	Yes
Thresholding	P-value threshold	Yes	NA	No
Lassosum	λ: LASSO penalty, s: R regularization	Yes	Equal	No
LDpred2-auto	p: Proportion of causal variants, h^2: Heritability	No	Equal	No
LDpred2-grid-sp	p: Proportion of causal variants h^2: Heritability	Yes	Equal	No
PANPRS	λ_0: LASSO penalty, λ_1: Traits total effect penalty	Yes	Equal	Yes
mvL[b]	λ: LASSO penalty, s: R regularization	Yes	Equal or BLD-X[c]	No

[a]: Clumping and thresholding

[b]: Multivariate Lassosum

[c]: Baseline linkage disequilibrium model-cross trait

Table S3: Predictive performance of PRSs on psychiatric traits in the Eastern Quebec schizophrenia and bipolar disorder study

SS[a]	Trait	n	Method	OR (95% CI)[b]	P-value	R^{2c}	$R^{2c}_{\text{liab.}}$	AUC
SZ[h]	SZ	Cases = 124 NAARs[d] = 442	C+T[e]	1.04 (0.85-1.28)	0.679	3×10^{-4}	3×10^{-4}	0.516
			Thresholding	1.01 (0.83-1.22)	0.960	0.000	0.000	0.497
			Lassosum	1.19 (0.95-1.49)	0.126	0.006	0.004	0.563
			LDpred2-auto	1.35 (1.10-1.67)	0.005	0.018	0.012	0.573
			LDpred2-grid-sp	1.43 (1.14-1.80)	0.002	0.025	0.016	0.595
			PANPRS	0.95 (0.77-1.17)	0.649	5×10^{-4}	3×10^{-4}	0.486
			mvL[f]	1.90 (1.52-2.38)	2×10^{-8}	0.079	0.051	0.685
			mvL(BLD-X[g])	1.85 (1.46-2.34)	3×10^{-7}	0.074	0.047	0.682
	BD	Cases = 205 NAARs = 442	C+T	1.21 (1.01-1.44)	0.041	0.008	0.005	0.558
			Thresholding	1.21 (1.02-1.43)	0.025	0.008	0.006	0.546
			Lassosum	1.11 (0.93-1.32)	0.261	0.003	0.002	0.549
			LDpred2-auto	1.39 (1.17-1.65)	2×10^{-4}	0.023	0.016	0.590
			LDpred2-grid-sp	1.36 (1.13-1.63)	9×10^{-4}	0.022	0.015	0.596
			PANPRS	1.08 (0.91-1.27)	0.381	0.001	9×10^{-4}	0.522
			mvL	1.44 (1.20-1.73)	7×10^{-5}	0.032	0.021	0.618
			mvL(BLD-X)	1.48 (1.24-1.77)	2×10^{-5}	0.035	0.024	0.620
	SAD	Cases = 35 NAARs = 442	C+T	1.83 (1.21-2.79)	0.005	0.019	0.025	0.626
			Thresholding	1.66 (1.16-2.38)	0.006	0.015	0.023	0.633
			Lassosum	1.58 (1.12-2.21)	0.008	0.009	0.016	0.616
			LDpred2-auto	1.59 (1.08-2.34)	0.019	0.019	0.021	0.604
			LDpred2-grid-sp	1.74 (1.16-2.62)	0.007	0.026	0.028	0.622
			PANPRS	1.19 (0.82-1.72)	0.353	0.002	0.003	0.573
			mvL	1.60 (1.12-2.29)	0.011	0.025	0.026	0.609
			mvL(BLD-X)	1.49 (0.99-2.22)	0.054	0.025	0.019	0.591
BD	SZ	Cases = 124 NAARs = 442	C+T	1.27 (1.03-1.56)	0.023	0.009	0.008	0.573
			Thresholding	1.18 (0.97-1.45)	0.104	0.005	0.004	0.547
			Lassosum	1.25 (1.00-1.55)	0.046	0.008	0.006	0.578
			LDpred2-auto	1.17 (0.96-1.44)	0.118	0.005	0.004	0.539
			LDpred2-grid-sp	1.27 (1.03-1.56)	0.024	0.011	0.008	0.568
			PANPRS	0.98 (0.80-1.19)	0.824	8×10^{-5}	7×10^{-5}	0.497
			mvL	1.47 (1.19-1.81)	3×10^{-4}	0.030	0.021	0.611
			mvL(BLD-X)	1.58 (1.27-1.96)	3×10^{-5}	0.041	0.028	0.631
	BD	Cases = 205 NAARs = 442	C+T	1.49 (1.25-1.78)	7×10^{-6}	0.032	0.023	0.614
			Thresholding	1.59 (1.33-1.90)	2×10^{-7}	0.044	0.031	0.623
			Lassosum	1.21 (1.01-1.43)	0.034	0.007	0.005	0.563
			LDpred2-auto	1.95 (1.63-2.33)	5×10^{-13}	0.094	0.060	0.682
			LDpred2-grid-sp	2.02 (1.68-2.42)	8×10^{-14}	0.097	0.062	0.686
			PANPRS	0.98 (0.83-1.16)	0.837	7×10^{-5}	5×10^{-5}	0.491
			mvL	2.06 (1.72-2.47)	7×10^{-15}	0.106	0.068	0.695
			mvL(BLD-X)	2.19 (1.82-2.63)	1×10^{-16}	0.123	0.077	0.713
	SAD	Cases = 35 NAARs = 442	C+T	1.65 (1.16-2.34)	0.005	0.017	0.025	0.633
			Thresholding	1.54 (1.09-2.15)	0.013	0.010	0.018	0.643
			Lassosum	1.60 (1.09-2.35)	0.015	0.014	0.021	0.645
			LDpred2-auto	1.81 (1.29-2.53)	6×10^{-4}	0.025	0.036	0.665
			LDpred2-grid-sp	2.21 (1.54-3.17)	2×10^{-5}	0.049	0.061	0.711
			PANPRS	0.74 (0.53-1.04)	0.086	0.005	0.009	0.414
			mvL	2.62 (1.83-3.74)	1×10^{-7}	0.080	0.092	0.759
			mvL(BLD-X)	2.51 (1.78-3.53)	1×10^{-7}	0.076	0.088	0.741

[a]: SS: summary statistics
[b]: Odds ratio of trait and 95% confidence interval for an increase of 1 standard deviation in PRS
[c]: Squared Pearson correlation ($\boldsymbol{R^2}$) and coefficient of determination on the liability scale ($\boldsymbol{R^2_{\text{liab.}}}$) [1] of PRS and diagnosis status. Population prevalence was set to 1% for SZ, 2% for BD and 0.3% for SAD [2]
[d]: Non-affected adult relatives
[e]: Clumping (window size=250 kb, r^2 and p selected by validation) and thresholding (p selected by validation)
[f]: Multivariate Lassosum
[g]: BLD-X: Baseline linkage disequilibrium model-cross trait
[h]: SZ: schizophrenia, BD: bipolar disorder, SAD: schizoaffective disorder

Table S4: Predictive performance of PRS on bipolar disorder with and without psychosis in the Eastern Quebec schizophrenia and bipolar disorder study

SS[a]	Trait	n	Method	OR (95% CI)[b]	P-value	R^{2c}	$R^{2c}_{\text{liab.}}$	AUC
SZ[h]	BD (with psychosis)	Cases = 93 NAARs[d] = 442	C+T[e]	0.92 (0.74-1.15)	0.473	0.001	0.001	0.489
			Thresholding	0.99 (0.80-1.23)	0.959	0.000	0.000	0.489
			Lassosum	0.88 (0.71-1.10)	0.266	0.003	0.002	0.496
			LDpred2-auto	1.31 (1.04-1.64)	0.022	0.009	0.009	0.569
			LDpred2-grid-sp	1.15 (0.90-1.46)	0.259	0.003	0.003	0.545
			PANPRS	0.96 (0.76-1.20)	0.695	3×10^{-4}	3×10^{-4}	0.487
			mvL[f]	1.40 (1.09-1.78)	0.008	0.020	0.015	0.609
			mvL(BLD-X[g])	1.45 (1.13-1.87)	0.004	0.026	0.019	0.618
	BD (without psychosis)	Cases = 112 NAARs = 442	C+T	1.62 (1.24-2.1)	4×10^{-4}	0.033	0.023	0.615
			Thresholding	1.42 (1.14-1.78)	0.002	0.023	0.017	0.593
			Lassosum	1.40 (1.10-1.78)	0.009	0.017	0.013	0.593
			LDpred2-auto	1.47 (1.17-1.85)	0.001	0.026	0.019	0.607
			LDpred2-grid-sp	1.62 (1.26-2.09)	2×10^{-4}	0.038	0.027	0.639
			PANPRS	1.19 (0.97-1.46)	0.087	0.005	0.004	0.550
			mvL	1.50 (1.20-1.87)	4×10^{-4}	0.027	0.022	0.625
			mvL(BLD-X)	1.52 (1.22-1.88)	2×10^{-4}	0.027	0.023	0.621
BD	BD (with psychosis)	Cases = 93 NAARs = 442	C+T	1.41 (1.11-1.79)	0.005	0.017	0.014	0.602
			Thresholding	1.59 (1.25-2.03)	2×10^{-4}	0.035	0.027	0.622
			Lassosum	1.03 (0.81-1.30)	0.815	1×10^{-4}	1×10^{-4}	0.522
			LDpred2-auto	2.03 (1.60-2.57)	6×10^{-9}	0.085	0.063	0.688
			LDpred2-grid-sp	2.00 (1.57-2.54)	2×10^{-8}	0.079	0.058	0.681
			PANPRS	1.00 (0.80-1.25)	0.986	0.000	0.000	0.496
			mvL	2.09 (1.65-2.66)	2×10^{-9}	0.090	0.067	0.699
			mvL(BLD-X)	2.23 (1.75-2.85)	8×10^{-11}	0.108	0.079	0.718
	BD (without psychosis)	Cases = 112 NAARs = 442	C+T	1.57 (1.27-1.93)	2×10^{-5}	0.028	0.025	0.623
			Thresholding	1.60 (1.29-1.98)	2×10^{-5}	0.031	0.027	0.625
			Lassosum	1.40 (1.14-1.74)	0.002	0.015	0.014	0.597
			LDpred2-auto	1.90 (1.52-2.38)	2×10^{-8}	0.064	0.050	0.676
			LDpred2-grid-sp	2.04 (1.62-2.55)	7×10^{-10}	0.071	0.057	0.690
			PANPRS	0.97 (0.78-1.20)	0.764	2×10^{-4}	2×10^{-4}	0.487
			mvL	2.03 (1.63-2.53)	2×10^{-10}	0.075	0.060	0.691
			mvL(BLD-X)	2.13 (1.71-2.66)	2×10^{-11}	0.085	0.067	0.709

[a]: SS: summary statistics
[b]: Odds ratio of trait and 95% confidence interval for an increase of 1 standard deviation in PRS
[c]: Squared Pearson correlation ($\boldsymbol{R^2}$) and coefficient of determination on the liability scale ($\boldsymbol{R^2_{\text{liab.}}}$)[1] for PRS and diagnosis status. Population prevalence was set to 0.9% for BD with psychosis and 1.1% for BD without psychosis (proportional to sample frequencies)
[d]: Non-affected adult relatives
[e]: Clumping (window size=250 kb, r^2 and p selected by validation) and thresholding (p selected by validation)
[f]: Multivariate Lassosum
[g]: BLD-X: Baseline linkage disequilibrium model-cross trait
[h]: SZ: schizophrenia, BD: bipolar disorder

References

[1] Lee, S. H., Goddard, M. E., Wray, N. R., and Visscher, P. M. (2012). A better coefficient of determination for genetic profile analysis. Genet Epidemiol 36, 214–24.

[2] Perälä, J., Suvisaari, J., Saarni, S. I., Kuoppasalmi, K., Isometsä, E., Pirkola, S., Partonen, T., Tuulio-Henriksson, A., Hintikka, J., Kieseppä, T. et al. (2007). Lifetime prevalence of psychotic and bipolar I disorders in a general population. Arch Gen Psychiatry 64, 19–28.
