# Supplementary material for: Multivariate extension of penalized regression on summary statistics to construct polygenic risk scores for correlated traits
Source: HGG Adv. 2023 May 20;4(3):100209. doi: 10.1016/j.xhgg.2023.100209 (PMC10276147; doi:10.1016/j.xhgg.2023.100209)
Supplement: Document S2. Article plus supplemental information [file mmc2.pdf]

HGG Advances

ARTICLE

Multivariate extension of penalized regression on summary statistics to construct polygenic risk scores for correlated traits

Meriem Bahda,[1,2] Jasmin Ricard,[2] Simon L. Girard,[2,3] Michel Maziade,[2,4] Maripier Isabelle,[2,5] and Alexandre Bureau[2,6,7,*]

Summary

Genetic correlations between human traits and disorders such as schizophrenia (SZ) and bipolar disorder (BD) diagnoses are well established. Improved prediction of individual traits has been obtained by combining predictors of multiple genetically correlated traits derived from summary statistics produced by genome-wide association studies, compared with single trait predictors. We extend this idea to penalized regression on summary statistics in Multivariate Lassosum, expressing regression coefficients for the multiple traits on single nucleotide polymorphisms (SNPs) as correlated random effects, similarly to multi-trait summary statistic best linear unbiased predictors (MT-SBLUPs). We also allow the SNP contributions to genetic covariance and heritability to depend on genomic annotations. We conducted simulations with two dichotomous traits having polygenic architecture similar to SZ and BD, using genotypes from 29,330 subjects from the CARTaGENE cohort. Multivariate Lassosum produced polygenic risk scores (PRSs) more strongly correlated with the true genetic risk predictor and had better discrimination power between affected and non-affected subjects than previously published sparse multi-trait (PANPRS) and univariate (Lassosum, sparse LDpred2, and the standard clumping and thresholding) methods in most simulation settings. Application of Multivariate Lassosum to predict SZ, BD, and related psychiatric traits in the Eastern Quebec SZ and BD kindred study revealed associations with every trait stronger than those obtained with univariate sparse PRSs, particularly when heritability and genetic covariance depended on genomic annotations. Multivariate Lassosum thus appears promising to improve prediction of genetically correlated traits with summary statistics for a selected subset of SNPs.

Introduction

Most prevalent human disorders have a polygenic component; i.e., a large number of genetic variants are involved in their etiology, each accounting for only a small percentage of the phenotypic variance. A polygenic risk score (PRS), usually defined as a weighted sum of single nucleotide polymorphism (SNP) alleles,[1] can stratify subjects with various levels of genetic risk for a disorder.[2] However, the accuracy of prediction of individual risk remains limited for most traits (see, e.g., Figure 4 of Zhang et al.[1]). One avenue to improve prediction accuracy is to take advantage of pleiotropy: the involvement of some genetic variants in multiple traits, which gives rise to well-established genetic correlations between human traits such as schizophrenia (SZ) and bipolar disorder (BD) diagnoses.[3,4] Indeed, combining predictors of multiple genetically correlated traits derived from summary statistics produced by genome-wide association studies (GWASs) under a mixed model framework[5] or a hierarchical Bayesian model[6] achieved improved prediction of individual traits compared with single-trait predictors.

The best linear unbiased predictors (BLUPs) derived from linear mixed models and their summary statistics versions SBLUP and MT-SBLUP[5] are PRSs where all SNPs contribute to the prediction. Since only a certain number of variants are involved in any disorder, the coefficients of a large proportion of SNPs are likely to represent only noise under this approach. Alternative approaches select SNPs to be included in PRSs defined from summary statistics. Hu et al.[6] proposed a hierarchical Bayesian model with a mixture distribution for the effect sizes where the effect of non-causal SNPs is a point mass probability at 0. Their PleioPred package is however limited to two traits, the Markov chain Monte Carlo runs required to estimate the posterior expectation of the SNP coefficients require long computing times, and these expectations never shrink to 0. Shrinking the coefficients of some SNPs to 0, essentially removing them from the PRS, is achieved via penalized regression with a LASSO (least absolute shrinkage and selection operator) penalty extended to summary statistics.[7,8] While the Lassosum package of Mak et al.[7] is restricted to single traits, the PANPRS package of Chen et al.[8] handles multiple traits by adding a second penalty term on the log of the sum of the absolute value of coefficients for the same SNP to favor including SNPs with large total effects. It does not however model correlation of SNP

[1]Department of Mathematics and Statistic, Laval University, Québec, QC G1V 0A6, Canada; [2]CERVO Brain Research Centre, Québec, QC G1E 1T2, Canada; [3]Department of Fundamental Sciences, University of Quebec in Chicoutimi, Chicoutimi, QC G7H 2B1, Canada; [4]Department of Psychiatry and Neurosciences, Laval University, Québec, QC G1V 0A6, Canada; [5]Department of Economics, Laval University, Québec, QC G1V 0A6, Canada; [6]Department of Social and Preventive Medicine, Laval University, Québec, QC G1V 0A6, Canada
[7]Lead contact
*Correspondence: alexandre.bureau@fmed.ulaval.ca
https://doi.org/10.1016/j.xhgg.2023.100209.
© 2023 The Author(s). This is an open access article under the CC BY license (http://creativecommons.org/licenses/by/4.0/).

effects. Our first objective was to extend LASSO-penalized regression with summary statistics to a model of correlated effects of SNPs on multiple traits.

Zhang et al.[1] noted that most PRS definitions including the BLUP and Lassosum assume that every SNP has the same contribution to the heritability of the trait, which implies an inverse relationship between the SNP minor allele frequency (MAF) and its expected effect on a quantitative trait or the risk of a disorder when genotypes are standardized. Speed et al.[9] showed that this model is usually unrealistic for human traits, and models allowing heritability contributions to depend on genomic annotations and more flexible relationships with MAF fit summary statistics data better. Hu et al.[6] and Zhang et al.[1] adapted several PRS definitions to base the contribution of each SNP on a heritability model and showed that using a model fitting GWAS data adequately improved the predictive performance of all the PRS definitions evaluated. Our second objective was to incorporate this idea into our multi-trait PRS, which involves applying annotation-based models to genetic covariances in addition to the heritabilities of the traits. By contrast, Hu et al.[6] applied annotation-based models to heritabilities only.

Our Multivariate Lassosum approach is implemented in the R package multivariateLassosum extending the Lassosum package and retaining its convenient data handling features. We evaluate its performance and compare it to the performance of approaches involving SNP selection: the multi-trait PANPRS[8] (attempts to apply PleioPred with sparse model[6] failed), and the single-trait Bayesian approach LDpred2[10] and standard clumping and thresholding (C + T) approach[11] in an extensive simulation study. Application of Multivariate Lassosum to predict SZ and BD, two traits with a well-established genetic correlation,[4] is illustrated in the Eastern Quebec SZ and BD kindred study.

Materials and methods

Multivariate trait model

We denote the number of phenotypes by q, the number of SNP markers by p, and the number of subjects by n. Our starting point is the linear mixed model of Maier et al.[5]:

$$y_{nq} = \boldsymbol{X}_{nq \times pq}\beta_{pq} + \varepsilon_{nq}, \qquad \text{(Equation 1)}$$

where y is a length nq vector of centered traits defined as $y = (y_{11}, .., y_{1q}, y_{21}, .., y_{2q}, .., y_{n1}, \ldots, y_{nq})^\top$, and $\boldsymbol{X}$ is a matrix of standardized SNP genotypes defined as

$$\boldsymbol{X} = \begin{pmatrix} \boldsymbol{X}_{11} & \boldsymbol{X}_{12} & \ldots & \boldsymbol{X}_{1p} \\ \boldsymbol{X}_{21} & \boldsymbol{X}_{22} & \ldots & \boldsymbol{X}_{2p} \\ \vdots & \vdots & & \vdots \\ \boldsymbol{X}_{n1} & \boldsymbol{X}_{n2} & \cdots & \boldsymbol{X}_{np} \end{pmatrix} \text{with } \boldsymbol{X}_{ij} = \begin{pmatrix} x_{ij} & 0 & \ldots & 0 \\ 0 & x_{ij} & \ldots & 0 \\ \vdots & \vdots & & \vdots \\ 0 & 0 & \cdots & x_{ij} \end{pmatrix},$$

where x_{ij} is the standardized genotype of subject i for the SNP j defined as $x_{ij} = (w_{ij} - 2p_j)/\sqrt{2p_j(1-p_j)}$ with w_{ij} the number of minor alleles (0, 1, or 2) for the ith individual at the jth SNP and p_j the empirical MAF. The standardized genotype x_{ij} is the same for all traits, since the SNP genotype does not vary by trait. The vector β of length pq is defined as $\beta = (\beta_{11}, .., \beta_{1q}, \beta_{21}, .., \beta_{2q}, .., \beta_{p1}, \ldots, \beta_{pq})^\top$, where β_{jk} is the genetic effect of the SNP j for the trait k. Maier et al.[5] assumed a constant variance-covariance matrix across SNPs, which implies an inverse relationship between the expected absolute effect of a copy of the minor allele and the MAF, a common assumption.[1] Given the evidence from Speed et al.[9] that this assumption does not hold for a vast array of common human traits, we allow marker-specific covariance matrices, such that $\beta_j \sim N(0, \boldsymbol{\Sigma}_{bj})$ where $\beta_j = (\beta_{j1}, .., \beta_{jq})$. We describe below how $\boldsymbol{\Sigma}_{bj}$ can be derived from a heritability model. ε represents a random error vector with $E(\varepsilon) = 0$ and $Var(\varepsilon) = \boldsymbol{\Sigma}_e$ where $\boldsymbol{\Sigma}_e = I_n \otimes \boldsymbol{\Sigma}_s$ is a $nq \times nq$ diagonal residual matrix, $\boldsymbol{\Sigma}_s = \text{diag}(\sigma_\varepsilon^2)$ is a diagonal $q \times q$ matrix, and $\sigma_\varepsilon^2 = (\sigma_{\varepsilon_1}^2, \ldots, \sigma_{\varepsilon_q}^2)$. In the initial derivation of the estimates from individual-level data, genotypes and traits are assumed to be measured on the same subjects. As we move later to summary statistics, genotypes and traits will be allowed to be measured in different samples of subjects of different sizes. When different traits are measured in different subjects, it is impossible to estimate residual covariances between traits. Following Maier et al.,[5] we set the residual covariances to 0.

Using C. R. Henderson's expression for the logarithm of the joint probability density function of y and β, (see for instance Jiang,[12] Equation 2.36), we can derive the log likelihood of model (1):

$$c - \frac{1}{2}\left[(y - \boldsymbol{X}\beta)^\top \boldsymbol{\Sigma}_e^{-1}(y - \boldsymbol{X}\beta) + \sum_j \beta_j^\top \boldsymbol{\Sigma}_{bj}^{-1}\beta_j\right].$$

We follow a similar methodology as Mak et al.[7] to derive estimates of β using the LASSO penalty,[13] further allowing the penalty to be weighted to implement adaptive LASSO.[14] We therefore minimize the following objective function:

$$\begin{aligned} f(\beta) &= (y - \boldsymbol{X}\beta)^\top \boldsymbol{\Sigma}_e^{-1}(y - \boldsymbol{X}\beta) + \sum_j \beta_j^\top \boldsymbol{\Sigma}_{bj}^{-1}\beta_j + 2\lambda \parallel W\beta\|_1^1 \\ &= y^\top \boldsymbol{\Sigma}_e^{-1} y - 2\sum_j \beta_j^\top \boldsymbol{\Sigma}_s^{-1} \sum_i \boldsymbol{X}_{ij} y_i \\ &\quad + \sum_j \left\{ \sum_l \left[\left(\sum_i \boldsymbol{X}_{ij}^\top \boldsymbol{X}_{il} \right) \beta_j^\top \boldsymbol{\Sigma}_s^{-1} \beta_l \right] + \beta_j^\top \boldsymbol{\Sigma}_{bj}^{-1} \beta_j \right\} \\ &\quad + 2\lambda \parallel W\beta\|_1^1, \end{aligned} \qquad \text{(Equation 2)}$$

where the summation indices, j and l, represent the SNPs, while the summation index i denotes the subjects, and $W = \text{diag}(w_{11}, .., w_{1q}, w_{21}, .., w_{2q}, .., w_{p1}, \ldots, w_{pq})$ represents the penalty weights.

As in Maier et al.,[5] we apply the proposed model to quantitative as well as dichotomous traits, which is also generally done with single traits because estimates of the parameters of this model can be obtained from summary statistics: correlations between $\boldsymbol{X}$ and y and among the genotypes of SNPs in $\boldsymbol{X}$. Chen et al.[8] started from the quadratic approximation of a LASSO-penalized logistic regression objective function and formally stated a series of approximations assuming small SNP effect sizes to recover the coordinate descent algorithm for the LASSO-penalized linear model based on summary statistics. When including the density of β in the expressions resulting from Chen et al.'s[8] approximations, we do not recover the coordinate descent algorithm presented in the next subsection. However, Speed and Balding[15] provided empirical evidence that p values from logistic and linear models are sufficiently similar when SNP effects are small to limit the impact of using logistic regression p values from GWAS for conversion into correlations, providing support for the application of the present model to dichotomous traits.

Estimation method

Using a notation similar to Mak et al.,[7] we denote by $r_k = \frac{1}{n}\boldsymbol{X}_{(1)}^T y_k$, the SNP-wise correlation between the SNPs and the trait k, and by $\boldsymbol{R} = \frac{1}{n}\boldsymbol{X}_{(1)}^T\boldsymbol{X}_{(1)}$, the linkage disequilibrium (LD) matrix, a matrix of correlations between SNPs, with y_k being the vector for trait k and $\boldsymbol{X}_{(1)}$ being the genotype matrix for a single trait. The vectors r_k can be approximated from publicly available summary statistics such as estimates of β coefficients and their standard errors or p values and sign of association test statistics as in Mak et al.[7] Such statistics may have been derived from linear regression for quantitative traits or logistic regression for dichotomous traits. $\boldsymbol{R}$ can be obtained from publicly available genotype reference databases.

Equation 2 then becomes the following:

$$f(\beta) = y^\top \boldsymbol{\Sigma}_e^{-1} y - 2\sum_j \beta_j^\top \boldsymbol{\Sigma}_s^{-1} n r_j + \sum_j\sum_l \beta_j^\top n \boldsymbol{R}_{jl}\boldsymbol{\Sigma}_s^{-1}\beta_l + \sum_j \beta_j^\top \boldsymbol{\Sigma}_{bj}^{-1}\beta_j + 2\lambda \parallel W\beta\parallel_1^1 . \quad \text{(Equation 3)}$$

However, since the genotype matrices $\boldsymbol{X}$ used to estimate $\boldsymbol{R}$ and r will in general be different, it is more accurate to write $\boldsymbol{R} = \frac{1}{n_r}\boldsymbol{X}_r^T\boldsymbol{X}_r$, where n_r is the number of subjects in the standardized genotype matrix $\boldsymbol{X}_r$ used to estimate LD. Furthermore, in practice, traits are measured in different subjects and in different numbers. It is therefore more accurate to consider n as a vector $n = (n_1, \ldots, n_k, \ldots, n_q)$ of the number of subjects in the sample used to compute the summary statistics for the q traits rather than a single integer. In this context, Mak et al.[7] noted that we are no longer in the framework of a penalized least squares problem and established that the following further regularization of the $\boldsymbol{R}$ matrix solves this issue. Replacing $\boldsymbol{R}$ with $\boldsymbol{R}_s = (1-s)r + s\boldsymbol{I}$ for some $0 < s < 1$, we can then rewrite Equation 3 as follows:

$$f(\beta) = y^\top \boldsymbol{\Sigma}_e^{-1} y - 2\sum_j \beta_j^\top \boldsymbol{\Sigma}_s^{-1}\mathrm{diag}(n) r_j + \sum_j \beta_j^\top \boldsymbol{\Sigma}_{bj}^{-1}\beta_j + \sum_j\sum_l \beta_j^\top \mathrm{diag}(n)\left(\tilde{\boldsymbol{X}}_j^\top \tilde{\boldsymbol{X}}_l + s\boldsymbol{I}\right)\boldsymbol{\Sigma}_s^{-1}\beta_l + 2\lambda \parallel W\beta\parallel_1^1,$$

where $\tilde{\boldsymbol{X}} = \sqrt{\frac{1-s}{n_r}}\boldsymbol{X}_r$. We estimate β assuming variances and covariances are known. In practice, estimates of variances and covariances from external sources need to be provided. Although the method allows for general genetic and residual variance terms, it is convenient to set the total variance to 1 and use heritability estimates $h_{gk} = \sum_{j=1}^p (\widehat{\boldsymbol{\Sigma}}_{bj})_{kk}, k = 1 \ldots q$ for the proportion of the total variance explained by additive genetic contributions. We then set $\widehat{\sigma}_\varepsilon^2 = (\widehat{\boldsymbol{\Sigma}}_s)_{kk} = 1 - h_{gk}, k = 1 \ldots q$. This only assumes in fact that the variances of all traits are equal, as multiplicative factors of the variances cancel in the solutions for the β coefficients below. Therefore, for SNP j and trait k, we find the β_{jk} estimate by minimizing the objective function:

$$f\left(\beta_{jk}\right) = \beta_{jk}^2\left[n_k\left(\tilde{\boldsymbol{X}}_j^\top \tilde{\boldsymbol{X}}_j + s\right)_k \widehat{\sigma}_{\varepsilon_k}^{-2} + \left(\widehat{\boldsymbol{\Sigma}}_{bj}^{-1}\right)_{kk}\right] - 2\times\beta_{jk}\left(-\frac{1}{2}\sum_{h\neq k}\left(\widehat{\boldsymbol{\Sigma}}_{bj}\right)_{kh}^{-1}\beta_{jh} + \left(\widehat{\boldsymbol{\Sigma}}_s^{-1}\right)_k n_k r_j - n_k\left(\widehat{\boldsymbol{\Sigma}}_s^{-1}\right)_{kk}\sum_{\ell\neq j}\tilde{\boldsymbol{X}}_j^\top\tilde{\boldsymbol{X}}_l\cdot\beta_l\right) + 2\lambda\left|w_{jk}\beta_{jk}\right|. \quad \text{(Equation 4)}$$

The minimization of $f(\beta_{jk})$ is an elastic net problem[16] with the covariance terms of the β added to the linkage disequilibrium terms of Mak et al.[7] in the quadratic penalty. Following a similar scheme as in Mak et al.,[7] the solution is found by iteratively updating β_{jk} as follows:

if < 0,

$$\beta_{jk}^{(t)} = \begin{cases} 0 & \text{if } A + \lambda w_{jk} > 0 \\ \dfrac{A + \lambda w_{jk}}{n_k\left(\tilde{\boldsymbol{X}}_j^\top\tilde{\boldsymbol{X}}_j + s\right)_k \widehat{\sigma}_{\varepsilon_k}^{-2} + \left(\widehat{\boldsymbol{\Sigma}}_{bj}^{-1}\right)_{kk}} & \text{else} \end{cases}$$

if > 0,

$$\beta_{jk}^{(t)} = \begin{cases} 0 & \text{if } A - \lambda w_{jk} < 0 \\ \dfrac{A - \lambda w_{jk}}{n_k\left(\tilde{\boldsymbol{X}}_j^\top\tilde{\boldsymbol{X}}_j + s\right)_k \widehat{\sigma}_{\varepsilon_k}^{-2} + \left(\widehat{\boldsymbol{\Sigma}}_{bj}^{-1}\right)_{kk}} & \text{else,} \end{cases}$$

where

$$A = \left(-\frac{1}{2}\sum_{h\neq k}\left(\widehat{\boldsymbol{\Sigma}}_{bj}\right)_{kh}^{-1}\beta_{jh}^{(t-1)} + \left(\widehat{\boldsymbol{\Sigma}}_s^{-1}\right)_k n_k r_j - n_k\left(\widehat{\boldsymbol{\Sigma}}_s^{-1}\right)_{kk}\sum_{\ell\neq j}\tilde{\boldsymbol{X}}_j^\top\tilde{\boldsymbol{X}}_l\cdot\beta_l^{(t-1)}\right).$$

For the weights w_{jk} we considered $w_{jk} = 1$, i.e., constant weight, $w_{jk} = \frac{1}{|\widehat{\beta}_{jk}|^\gamma}$ where $\widehat{\beta}_{jk}$ are the estimates from the GWAS, and γ is a tuning parameter (the original adaptive LASSO of Zou,[14] except that here $\widehat{\beta}_{jk}$ is in general inconsistent), and $w_{jk} = \frac{1}{|\widehat{\beta}_{mvL,jk}|}$, where $\widehat{\beta}_{mvL,jk}$ are the estimates from Multivariate Lassosum with $w_{jk} = 1$ (the proposal by Bühlmann and Geer[17]).

Selection of tuning parameters

The standard approach is to select tuning parameters in a validation set independent from the training set. Summary statistics are generally available only for the full sample on which GWASs have been conducted for each trait. We adopted the approach of Zhang et al.[1] to simulate pseudo summary statistics for a training and a validation sample from full-sample summary statistics, except we let the variance of the trait differ from 1. Thus, instead of estimating $\frac{1}{n_k}\boldsymbol{V}_k$, the covariance matrix of r_k, by $\frac{1}{n_r}\boldsymbol{X}_r^T\boldsymbol{X}_r$ as in Zhang et al.,[1] we instead use $\frac{1}{n_r}s^2(r_k)\boldsymbol{X}_r^T\boldsymbol{X}_r$ where $s^2(r_k)$ is the empirical variance of r_k. Let n_{Ak} and n_{Bk} be the size of the training and validation sets for trait k. In our notation, the SNP-wise correlation between SNPs and the trait k in the training set is obtained as $r_{Ak} = r_k + \sqrt{\frac{n_{Bk}}{n_{Ak}}}s(r_k)\frac{1}{\sqrt{n_r}}\boldsymbol{X}_r^\top g$, where g is a vector of n_r drawn from the standard Gaussian distribution, and $r_{Bk} = \frac{1}{n_{Bk}}(n_k r_k - n_{Ak} r_{Ak})$. In our application, we set $n_{Bk} = n_B$, 10% of the average of n for all traits, and $n_{Ak} = n_k - n_B$.

Following Mak et al.,[7] we select the value of a tuning parameter λ that maximizes the correlation between the PRS and y in a validation sample. Mak et al.[7] showed that this is equivalent to the value of λ maximizing the function:

$$f(\lambda) = \frac{\beta_\lambda^\top r_B}{\sqrt{\frac{1}{n_0}\beta_\lambda^\top \boldsymbol{X}_0^\top \boldsymbol{X}_0 \beta_\lambda}}, \quad \text{(Equation 5)}$$

where $r_B = (r_{B11}, .., r_{B1q}, r_{B21}, .., r_{B2q}, .., r_{Bp1}, \ldots, r_{Bpq})$, and $\boldsymbol{X}_0$ is a matrix of the standardized genotypes in a sample of n_0 subjects independent from the training sample in the same format as $\boldsymbol{X}$. The

range of values of λ was set such that the proportion of SNPs with $\beta_{jk} \neq 0$ represented at least 5% of all SNPs for every trait k.

Mak et al.[7] also proposed a pseudovalidation approach that does not require trait values or trait-genotype correlations in the validation set. It requires a shrunken estimate of the r, which can be calculated as

$$\hat{r}_{jk} = r_{jk} h_{jk},$$

where h_{jk} is the minimum of the posterior expected loss, which for a quantitative trait, Mak et al.[18] define as a quadratic loss. Generalization to a multivariate trait would require the posterior expectation of products of β coefficients for the different traits, which would involve unknown quantities. For dichotomous traits, considering a binomial log likelihood loss requires only the posterior expectation of the β coefficients and not of their products. We derived a solution for h_{jk} under such loss function and used it to implement pseudovalidation in our simulations, but performance was poor, so we do not present this solution.

In the analyses of real and simulated data with Lassosum and Multivariate Lassosum reported in this work, a selection procedure was applied only to the LASSO penalty λ parameter. The s regularization parameter was set to 0.5 following the observation of Mak et al.[7] that such a value of s tended to achieve the best performance. In a sensitivity analysis on simulated datasets, we also tried to set s to 0.2, 0.9, or 1.

Specification of the SNP effects covariance matrices

There is evidence that the expected heritability contributed by an SNP varies as a function of SNP characteristics for a large collection of traits, and several models have been proposed to explain it based on genomic annotations.[9] Recent developments in stratified LD score regression enable to estimate such models including continuous-valued annotations.[19] Shi et al.[20] applied one such model, the Baseline-LD-X (BLD-X) model, to the trans-ethnic genetic covariance of one trait in two ethnically distinct populations. Here we apply it to the genetic covariance between two traits in the same population. That is, we express the expectation of the product of Z scores Z_{j1} and Z_{j2} for two traits in the same population as

$$E[Z_{j1}Z_{j2}] = \sqrt{n_1 n_2}\sum_{C}\ell(j,C)\theta_C + \sqrt{n_1 n_2}b_{12}, \qquad \text{(Equation 6)}$$

where $\ell(j,C) = \sum a_{lC}R_{jl}^2$ is the usual LD score of SNP j with respect to annotation C^ltaking value a_{lC} for SNP l instead of the trans-ethnic score of Shi et al.,[20] and θ_C is thus the effect of annotation C on the genetic covariance of the two traits. We add to the model of Shi et al.[20] the intercept term b_{12} to account for potential sample overlap, but we fit the model with $b_{12} = 0$ unless noted otherwise. We fit the model to Z_{j1} and Z_{j2} from summary statistics for traits 1 and 2 to estimate the θ_C as well as the effects $\tau_{kC}, k = 1,2$ of the annotations on the heritabilities. The expected heritabilities and genetic covariance contributed by each SNP j are then predicted using the values of their annotations:

$$\hat{h}_{gk}^2(j) = \sum_C a_{jC}\hat{\tau}_{kC}, k = 1,2 \text{ and } \hat{\rho}_g(j) = \sum_C a_{jC}\hat{\theta}_C,$$

and we set

$$\boldsymbol{\Sigma}_{bj} = \begin{pmatrix} \hat{h}_{g1}^2(j) & \hat{\rho}_g(j) \\ \hat{\rho}_g(j) & \hat{h}_{g2}^2(j) \end{pmatrix}.$$

Simulation framework

We simulated summary statistics for two genetically correlated traits to compare the predictive performance of the different options of Multivariate Lassosum against alternative PRS construction methods involving SNP selection. The parameters that were varied in our simulations are summarized in Table 1.

We used actual genotype data on 29,330 subjects from the CARTaGENE research platform (www.cartagene.qc.ca)[21] genotyped with the Illumina Global Screening Array (GSA). We filtered out variants and participants using the following criteria: genotype missing rate > 0.01 and MAF < 0.001. We also only considered the autosomes. We then imputed the small number of remaining missing genotypes using the mode. There remained 423,552 SNPs and all 29,330 individuals. The genotype data was randomly divided into three samples: the first sample of 23,330 individuals was used to generate the summary statistics. The second sample of 3,000 individuals was used as a reference panel for PRS construction and as validation set for selecting penalty parameters. The third sample of 3,000 individuals was used to evaluate the predictive performance of the PRSs from all methods compared. To evaluate the method in smaller samples, we extracted the first three genotyping batches comprising 10,139 subjects. The numbers of subjects per sample are summarized for the full cohort of 29,330 and the reduced cohort of 10,139 in Table 1.

The two genetically correlated traits in our simulation study were inspired from SZ and BD. To obtain realistic contributions of SNPs to heritabilities and genetic covariance, we fitted the BLD-X model to the most recent SZ[22] and BD[23] summary statistics for the 250,652 SNPs with genotypes for which such statistics as well as LD scores and MAF were available for the European (EUR) 1000 Genomes Project sample and predicted $\hat{h}_{g1}^2(j), \hat{h}_{g2}^2(j)$ and $\hat{\rho}_g(j)$ for each SNP j. For this prediction, annotations were available for 419,492 SNPs; the other 4,060 SNPs were assigned the baseline category for dichotomous annotations and the mean value for SNPs on the same chromosome for continuous annotations.

SNP effects were simulated following the previously proposed model[24,6] that some proportion of SNPs have a causal effect on a trait and the rest of the SNPs have no effect, and there is overlap between the SNPs having a causal effect on each trait. The distribution of the effect of the causal SNPs depended on the assumed heritability model: either based on the predictions from the BLD-X model annotations as described above or constant across SNPs (resulting in a mixture of four genetic covariance matrices). Then, simulation parameters were varied as follows: for *heritability*, we set the variance of both traits to $\text{Var}(y_1) = \text{Var}(y_2) = 1$, and for a high observed-scale SNP heritability, we set the value for trait 1 to $h_{g1}^2 = 0.47$ and for trait 2 to $h_{g2}^2 = 0.45$, the estimates reported by Maier et al.[5] for SZ and BD based on the summary statistics available at the time (Psychiatric Genomic Consortium [PGC], version PGC2 for SZ and PGC1 for BD). For a low heritability, we set the observed-scale SNP heritabilities of the two traits to 0.10 and 0.09. For *correlation*, when we simulate a mixture of four genetic-covariance-matrices, we set the genetic correlation between the two traits to $r_g = 0.59$, the genetic correlation between SZ and BD presented in Maier et al.,[4] for a high correlation or $r_g = 0.44$ for a moderate correlation (same as for the BLD-X model below). For *polygenicity*, we then set the probability that an SNP has a causal effect on both traits 1 and 2 to 0.35 for high polygenicity or 0.08 for low polygenicity. We also set the variance explained by an SNP and solved for the probability that an SNP has a causal effect on trait 1 alone and on trait 2 alone to obtain

Table 1. Parameters of the simulation scenarios

Parameter	Reference value	Alternative value
Training, validation, and test sample sizes	23,330; 3,000; 3,000	8,139; 1,000; 1,000
Trait heritability (trait 1, trait 2)	high (0.47, 0.45)	low (0.10, 0.09)
Trait polygenicity (trait 1, trait 2)	high (0.49, 0.47)	low (0.12, 0.10)
Heritability model	derived from BLD-X model	four covariance matrices
Trait correlation with four covariance matrices	0.59	0.44
Sample overlap for the two traits (sensitivity)	none	inducing correlation of 0.16 and 0.32

the proportions of SNPs influencing each trait reported in Table 1. SNPs were then randomly assigned as causal for both traits, for one of the two or for neither according to probabilities reported in the simulation parameters section of the supplemental information.

We kept the same probabilities of causality and heritabilities when we made the heritabilities and genetic covariance of the two traits depend on predictions from the BLD-X model annotations, but the assignment of trait causality status was constrained by the predictions from the BLD-X model; i.e., SNPs with a predicted heritability ≤ 0 for either trait were set as non-causal for the trait in question in all replicates (see the simulation parameters section of the supplemental information for details and Table S1 on the proportions and numbers of SNPs for each combination of trait causality statuses). We then generated the vector (β_{j1}, β_{j2}) of genetic effects for the SNP j for traits 1 and 2 as follows:

$$\begin{pmatrix}\beta_{1j}\\ \beta_{2j}\end{pmatrix} \sim \begin{cases} N\left(\begin{pmatrix}0\\0\end{pmatrix}, \begin{pmatrix}\alpha_1 \widehat{h}^2_{g1}(j) & \alpha\widehat{\rho}_g(j)\\ \alpha\widehat{\rho}_g(j) & \alpha_2\widehat{h}^2_{g2}(j)\end{pmatrix}\right) & \text{if SNP } j \text{ causes traits 1 and 2}\\ \begin{pmatrix} N\left(0, \alpha_1\widehat{h}^2_{g1}(j)\right)\\ 0\end{pmatrix} & \text{if SNP } j \text{ causes trait 1}\\ \begin{pmatrix} 0\\ N\left(0, \alpha_2\widehat{h}^2_{g2}(j)\right)\end{pmatrix} & \text{if SNP } j \text{ causes trait 2}\\ \begin{pmatrix}0\\0\end{pmatrix} & \text{if SNP } j \text{ causes neither trait 1 nor trait 2,}\end{cases} \quad \text{(Equation 7)}$$

where α_1 and α_2 are scaling factors so that the heritability of traits 1 and 2 equal the prespecified values and $\alpha = \sqrt{\alpha_1\alpha_2}$. This scaling was required because only a fraction of all SNPs actually contributing to SZ and BD heritability were included in the simulations and because the specified heritabilities of traits 1 and 2 differ from the values from the BLD-X model on the most recent summary statistics of SZ and BD. We did not impose a prespecified genetic covariance and obtained a value of genetic correlation = 0.44.

For the scenario with a mixture of four genetic covariance matrices, (β_{j1}, β_{j2}) were generated as above with the matrix element values described in the simulation parameters section of the supplemental information. We then obtained the observed correlation coefficients for each genomic region l and each trait k using

$$r_{lk} \sim N(\widehat{\boldsymbol{R}}_l\beta_{lk}, \widehat{\boldsymbol{R}}_l / n_k), \quad \text{(Equation 8)}$$

where $\widehat{\boldsymbol{R}}_l$ is the observed correlation matrix of the lth region from the genotype $\boldsymbol{X}$, and n_k is the sample size assuming r_{l1} and r_{l2} were conditionally independent given their expected value. Here we point out that we did not generate the vector of genetic effects β by genomic regions like in Mak et al.[7] However, we generated the observed correlation coefficients by genomic regions, as shown in Equation 8. In a sensitivity analysis, we introduced correlation between r_{l1} and r_{l2} that could result from overlap in the samples used in the GWAS (e.g., common controls). We then have $Cov[r_{l1}, r_{l2}] = \rho_o\widehat{\boldsymbol{R}}_l/n_k$, where ρ_o was set to $\widehat{\rho_o} = \widehat{b}_{12}/\sqrt{\widehat{b}_1\widehat{b}_2}$, where $\widehat{b}_{12}$, $\widehat{b}_1$, and $\widehat{b}_2$ are the intercept term estimates from cross-trait and trait-specific Linkage Disequilibrium Score Regression fit of SZ and BD summary statistics, an approach inspired by Turley et al.[25] The cross-trait intercept term estimate was $\widehat{b}_{12} = 0.18$ leading to a between-trait summary-statistic correlation $\widehat{\rho_o} = 0.16$. We also simulated a scenario with more substantial sample overlap by doubling the between-trait summary-statistic correlation to $\widehat{\rho_o} = 0.32$.

For the subjects in the test set, the two dichotomous traits were simulated under a liability threshold model with the liability $L_{ik} = G_{ik} + E_{ik}$, where $G_{ik} = \sum_j \beta_{jk}\boldsymbol{X}_{ij}$ is the true standardized genetic predictor, the variance of the environmental component E_{ik} is set to $1 - h_{gk}$, and the thresholds for being affected were set such that traits 1 and 2 had prevalence 1% and 2%,

respectively. The area under the receiver operating curve (AUC) was computed in the test set for the two traits using the simulated traits and PRS from every method included in the comparison.

Methods compared

We compared Multivariate Lassosum with a constant genetic covariance matrix across SNPs and with genetic covariance matrices based on the predictions from BLD-X model annotations for each SNP, although in practice one would try both models and select the one performing best in the validation set. The matrices were scaled such that the total heritability and covariances equaled the estimates for SZ and BD. Hence, the constant matrices were set to

$$\boldsymbol{\Sigma}_b = \frac{1}{p}\begin{pmatrix} 0.47 & 0.27 \\ 0.27 & 0.45 \end{pmatrix},$$

and for the variable matrices, appropriate scaling factors were applied (these differed from α_1 and α_2 above as we do not force SNP effects to 0 like we do when we simulate the true values).

The results from Multivariate Lassosum were compared with those from a previously published multi-trait method, PANPRS,[8] and from single-trait methods incorporating SNP selection: the original Lassosum,[7] the sparse option of LDpred2,[10] and p value thresholding, the last two implemented in the bigsnpr R package. We attempted to apply PleioPred with sparse model[6] to our simulated data, but a NaN value was returned for subsets of beta coefficients despite correct matching of SNP identifiers. An exchange with PleioPred authors did not resolve this issue.

All methods were applied to the same simulated training samples. Tuning parameters of all methods were selected in the validation set, except for LDpred2 for which we used the auto option to automatically estimate the proportion of causal variants and the trait heritability from summary statistics data. This was done because LDpred2 requires individual traits in the validation set contrary to evaluating $f(\lambda)$ (Equation 5) as we do for Lassosum-derived approaches and p value thresholding, noting that the predictive performance of the auto option of LDpred2 matched that of the validation-based options.[10] The burn-in and run length of the Gibbs sampler of LDpred2 were set to their default values. The default set of heritability values was used, but the range of the proportion of causal variants was expanded from the default 0.0001–0.2 range to the 0.0001–0.5 range (0.0001, 0.0005, 0.001, 0.005, and 0.01, followed by a sequence of 25 equally spaced values from 0.05 to 0.5) to encompass our high polygenicity scenario. PRSs obtained from the resulting grid of hyperparameter values were processed as recommended by Privé et al.[10] A sequence of eight p value thresholds was used for thresholding and C + T methods: 1, 0.75, 0.5, 0.25, 0.1, 0.05, 0.001, and 1e-4. For the latter method, seven correlation thresholds were tested: 0.01, 0.05, 0.1, 0.2, 0.5, 0.8, and 0.95. For PANPRS, vectors of tuning parameter values were generated by applying the initial configuration steps to all autosomal SNPs, and then the generated vectors were passed to chromosome-specific analysis runs as recommended (personal communication with Dr. T.H. Chen). The methods compared, their parameters, and other features are summarized in Table S2.

Application to schizophrenia and bipolar disorder

Genetic correlation between SZ and BD has been established through familial coaggregation studies,[26] mixed model analysis on individual data,[4] as well as LD score regression on summary statistics.[3,5] We illustrate the gain in predictive power of Multivariate Lassosum over single-trait methods on these two traits using trait and genotype data from the SZ and BD Eastern Quebec kindred study.[27,28] The best-estimate lifetime DSM-IV diagnosis was made as outlined in previous reports.[29,30] Due to the presence of SZ, BD, and related diagnoses in the same sample, the risk of bias in favor of a particular diagnosis was minimized. Signed consent was obtained from all participants or from the parents for participants under 18, as reviewed by our University Ethics Committee. SNP array genotyping was performed in two waves using DNA extracted from immortalized lymphocytes or fresh blood by affinity column (Midi prep Qiagen). The same quality control criteria were applied to both waves (see genotyping quality control procedures in the supplemental information), leaving 1,120 genotyped subjects: 507 subjects genotyped at 622,184 autosomal SNPs with the Illumina Infinium Human OmniExpress array in the first wave[31] and 613 subjects genotyped at 502,425 SNPs with the Illumina GSA in the second wave. Before imputation, the phasing of our familial sample genotypes was done by Shapeit2 software, which allowed us to use the family information in the phasing process via the duoHMM algorithm.[32] Imputation of all common SNPs and indels was then made on the Michigan Imputation Server using the Haplotype reference consortium panel.[33] The large number of SNPs led us to select the SNPs with an MAF > 0.1, as was done before,[34] leaving 3,639,921 SNPs.

Since LDpred2 auto option does not require a validation set, LDpred2 auto was applied to the original univariate coefficients released by the PGC in their summary statistics ($n = 161{,}405$ for SZ, 413,466 for BD). For all other methods, we generated pseudo summary statistics for SZ and BD to mimic a validation set of size 28,744 and training sets of size 132,661 for SZ and 384,722 for BD by applying the approach described in materials and methods to the 6,398,847 SNPs with summary statistics for SZ[22] and BD[23] on the autosomal genome and with genotypes imputed in both the subjects genotyped using the OmniExpress array and using the GSA array. We used the genotypes of the EUR 1000 Genomes sample as X_r to generate the pseudo summary statistics. After selection of the tuning parameter values, the PRS coefficients of each method were re-estimated on the original summary statistics. We adapted the application of C + T and LDpred2 with a grid of hyperparameter values (including for LDpred2 the proportion of causal SNPs: LDpred2 grid-sp) to compute the selection criterion (Equation 5) on validation set pseudo summary statistics, instead of the usual approach of measuring predictive performance on the trait in the validation set. The large number of SNPs led us to perform an initial clumping to speed up PANPRS computations as recommended (personal communication with Dr. T.H. Chen). In order to obtain a common set of SNPs for SZ and BD, we assigned to each SNP the minimum of the association p value for SZ and BD. A clumping $r^2 = 0.5$ and a window size of 250 kb using Plink 1.9[35] led to the selection of 365,527 SNPs.

Among the 1,120 genotyped subjects, diagnoses were distributed as follows: 205 BD, 124 SZ, 35 schizoaffective disorder (SAD), 442 non-affected adult relatives (NAARs), and 314 relatives whose diagnosis was considered unknown (e.g., parents of affected subjects who were not themselves affected). BD subjects were evaluated for the presence of symptoms of psychosis, which were detected in 93 genotyped BD subjects. PRSs were standardized to have mean 0 and variance 1 in the NAARs group separately for the two genotyping arrays. Association between diagnosis and PRSs was evaluated under a logistic model estimated by

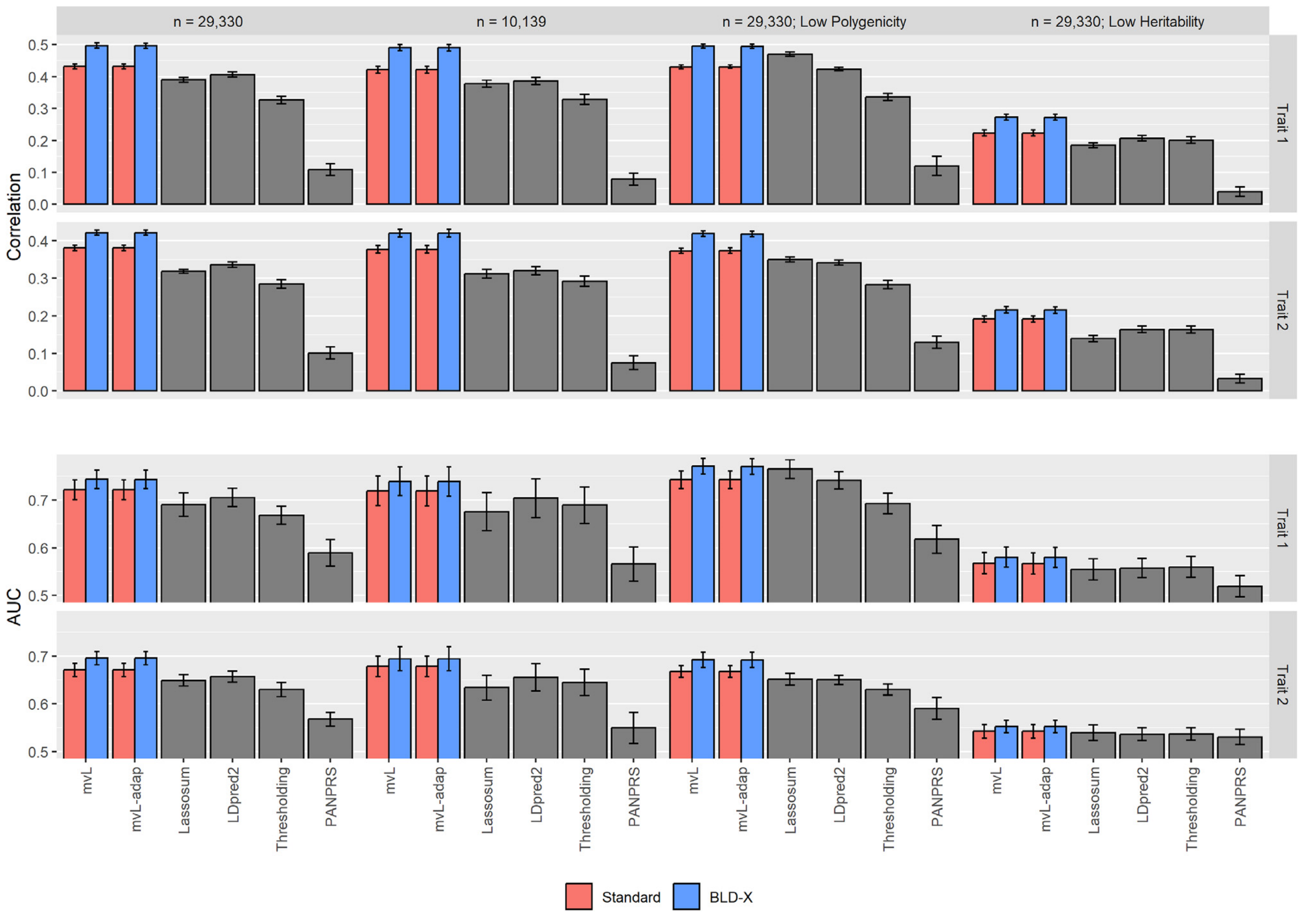

Figure 1. Comparison of PRS predictive performance for multiple variations of the simulation scenario where heritability and genetic covariance of two traits depend on genomic annotations

Top panel: Pearson correlation of the PRS with the true predictor. Bottom panel: area under the receiver operating curve (AUC) for the prediction of simulated traits by PRS. Mean and 95% confidence interval based on 20 replicates. The penalty parameter λ (for the penalized regression methods) and the threshold for thresholding were set to the values maximizing the correlation between the PRS and the trait y in a validation set. Methods compared: mvL: Multivariate Lassosum with constant penalty, mvL-adapt: Multivariate Lassosum with adaptive penalty based on the initial estimates from mvL. Models for heritability and covariance used in analysis : BLD-X: Baseline linkage disequilibrium model-cross trait; Standard: constant contribution of standardized genotypes of all SNPs.

generalized estimating equation with a variance estimator robust to familial dependence.

SNP-specific contributions to heritabilities and covariances were predicted from the BLD-X heritability model fitted to pseudo summary statistics for the 3,324,089 SNPs with LD scores and MAF available for the EUR sample using the approach described above. Annotations were available for 3,611,681 SNPs; values were imputed for the other 28,240 SNPs as for the simulations above. In this model fit, after setting negative SNP predicted heritabilities to 0, some predicted covariances led to non-semi-positive definite genetic covariance matrices, and these covariances were replaced by $\max(0, \sqrt{\widehat{h}^2_{g1}(j)\widehat{h}^2_{g2}(j) - 0.001})$, where $\widehat{h}^2_{g1}(j)$ is the predicted contribution of SNP j to SZ and $\widehat{h}^2_{g2}(j)$ to BD. The sum of the corrected contributions of the 3,639,921 SNPs were 0.85 for SZ, 0.17 for BD, and 0.12 for the covariance between SZ and BD (genetic correlation = 0.31). These values were used to define the constant covariance matrix Σ_b for the analysis with equal contribution of all SNPs. The sample of subjects genotyped with the OmniExpress array were used as reference panel for all PRS methods.

Results

Simulation study

The version of Multivariate Lassosum with the heritability model best fitting the simulated data (e.g., BLD-X for the simulation scenarios where heritability and genetic covariance depended on genomic annotations) applied to the two simulated traits generally achieved higher predictive performance than competing methods analyzing either both traits jointly or each trait separately in terms of estimated PRS vs. true predictor correlation and AUC (Figures 1 and S2) in the reference scenario and when reducing the sample size, correlation, or heritability. In the scenario with low polygenicity, the original Lassosum performed about as well as Multivariate Lassosum for trait 1. In this instance, the original Lassosum and LDpred2 achieved better performance than the version of Multivariate Lassosum with the less adapted heritability model. Under the other scenarios where the data were generated

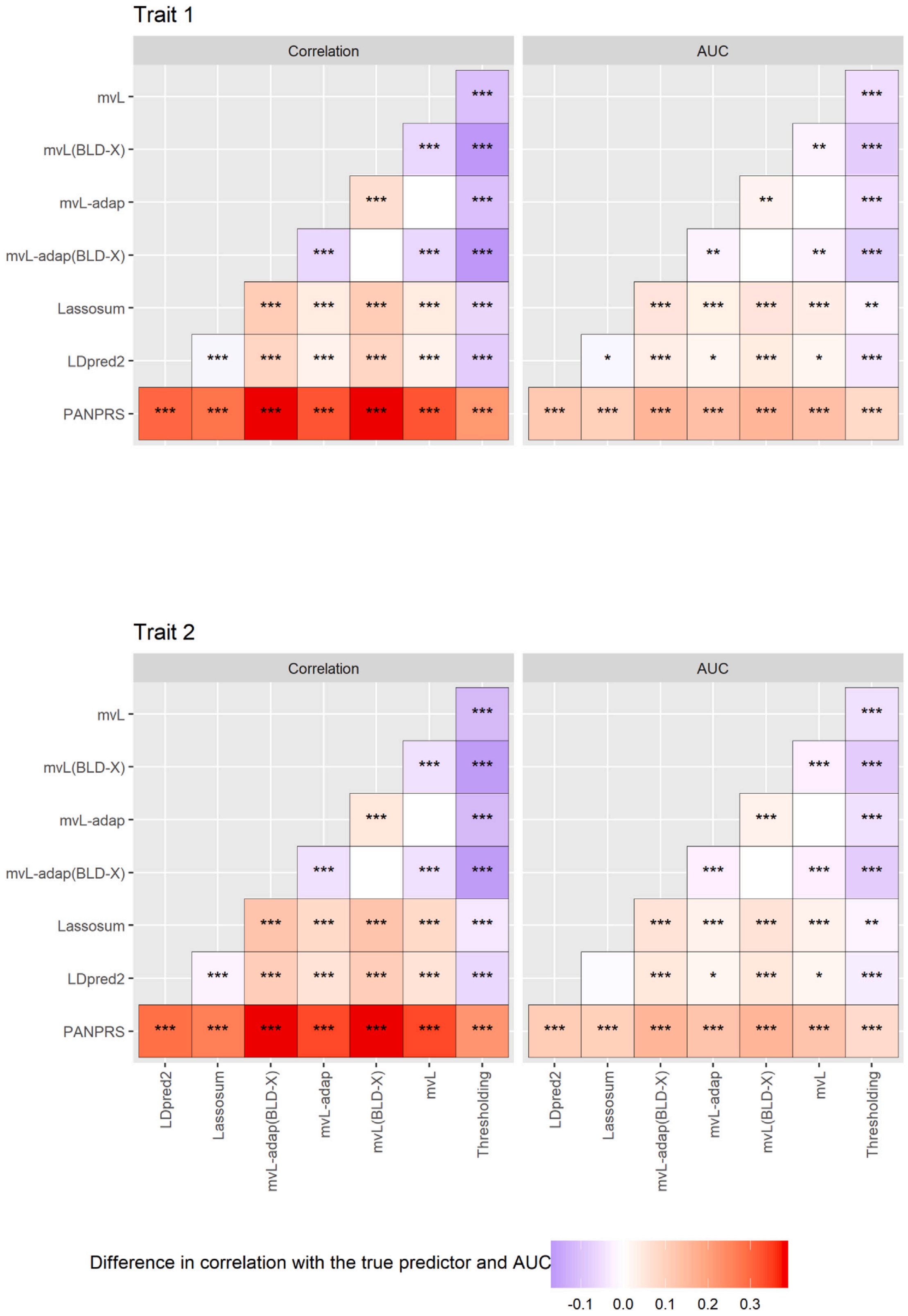

Figure 2. Difference in PRS predictive performance under the reference simulation scenario where heritability and genetic covariance of two traits depend on genomic annotations

Top panel: difference in Pearson correlation of the PRS with the true predictor between methods. Bottom panel: difference in area under the receiver operating curve (AUC) for the prediction of simulated traits by PRS. See legend of Figure 1 for the definitions of acronyms for the methods. Statistical significance of the difference was evaluated by paired t tests using 20 replicates, and p values were corrected for multiple testing using the Bonferroni method. $^{*}p < 0.05$, $^{**}p < 5 \times 10^{-4}$, $^{***}p < 5 \times 10^{-8}$.

Table 2. Mean run time (SD) in minutes per replicate for every method across the 20 replicates of the simulations using 29,330 subjects and 10,139 subjects

Methods	Cores used	n = 29,330; p = 423,552	n = 10,139; p = 479,158
mvL	80	11.86 (0.33)	7.32 (0.42)
mvL (BLD-X)	80	13.25 (0.31)	7.78 (0.11)
mvL-adap	80	11.94 (0.09)	7.41 (0.43)
mvL-adap (BLD-X)	80	12.24 (0.12)	7.47 (0.41)
Lassosum[a]	80	15.72 (0.04)	7.71 (0.91)
LDpred2[a]	40	23.25 (0.29)	22.01 (4.29)
PANPRS	1	2,992 (68)	3,834 (80)

[a]Cumulative time of two runs, one on each of the two traits.

with a mixture of four genetic covariance matrices, the original Lassosum and LDpred2 also outperformed Multivariate Lassosum with the BLD-X model. Although many of the above reported performance differences were small, Figures 2 and S2–S5 show that they were statistically significant.

The performances of Multivariate Lassosum and the other PRS methods were insensitive to the variation of most simulation parameters, except heritability. Correlation of summary statistics due to overlap of the samples for the two traits had also little impact on performance even when summary-statistic correlation was doubled compared with the estimate for SZ and BD (Figures S6 and S7). The fixed value 0.5 for the regularization parameter s led to optimal or near optimal performance of Multivariate Lassosum under the reference simulation scenario (Figure S8). Using an adaptive LASSO penalty did not improve the predictive performance over the initial analysis with a constant penalty for all coefficients. We show the results when adaptive weights were defined using the coefficient estimates from the Multivariate Lassosum analysis with constant penalty; results using the weight definition inspired by Zou[14] were nearly identical (not shown). Computing times on a multi-threaded computer cluster for a Multivariate Lassosum run were a little shorter than two runs of Lassosum for the two traits, with the BLD-X model requiring slightly more time than the constant genetic covariance matrix (Table 2). LDpred2 being limited to a single node, we could only request half the number of cores as the Lassosum-derived methods, while we were restricted to a single core for PANPRS. This led to consequently larger computing times. The requested random access memory of 10 Gb per core (20 Gb for PANPRS) is an upper bound on the actual memory used.

Analysis of schizophrenia and bipolar disorder

The selection criterion (Equation 5) evaluated on pseudo summary statistics of SZ and BD validation sets reached a higher value for Multivariate Lassosum PRSs under the BLD-X model than under a constant genetic covariance matrix. Although this implies selection of the BLD-X model, we report results from the application of both models to the test set in Figure 3 and Tables S3 and S4 to be able to compare them. Multivariate Lassosum PRSs explained a greater proportion of the variance of SZ, BD, and SAD and had better discrimination power between affected and non-affected subjects as measured by the AUC, and the odds ratios of these disorders for an increase of 1 SD in Multivariate Lassosum PRSs were larger than for competing methods in the Eastern Quebec SZ and BD kindred sample (Figure 3 and Table S3) when the PRS was defined based on GWAS of the same disorder (SZ with SZ and BD with BD) and in some cases when the PRS was defined based on other disorders (SZ and SAD with BD summary statistics and BD with SZ summary statistics). Odds ratios between quartiles of risk defined from Multivariate Lassosum PRSs tended to also be larger than for PRSs of other methods in the same instances, with more variability than the odds ratios for an increase of 1 SD (Figure S9). For SAD with SZ summary statistics, C + T performed best instead.

The BLD-X model slightly improved the proportion of explained variance, AUC, and odds ratio over the constant genetic covariance matrix for BD when predicting from SZ pseudo summary statistics and for SZ and BD when predicting from BD pseudo summary statistics, and the two models performed similarly for the other analyses where mvL performed best among the evaluated methods. The largest odds ratio, liability-scale R^2, and AUC were achieved for SAD and the largest observed-scale R^2 for BD, both with PRSs derived from BD pseudo summary statistics. Distinguishing BD with and without psychosis did not impact substantially the variance explained and odds ratios for PRSs derived from BD pseudo summary statistics, as well as for PRSs derived from SZ pseudo summary statistics (Table S4). The BLD-X model generally led to better performance than the constant genetic covariance matrix for BD with and without psychosis, irrespective of whether SZ or BD pseudo summary statistics were used. We repeated the analysis after fitting the BLD-X model with an intercept term in Equation 6 and the results remained almost identical (not shown).

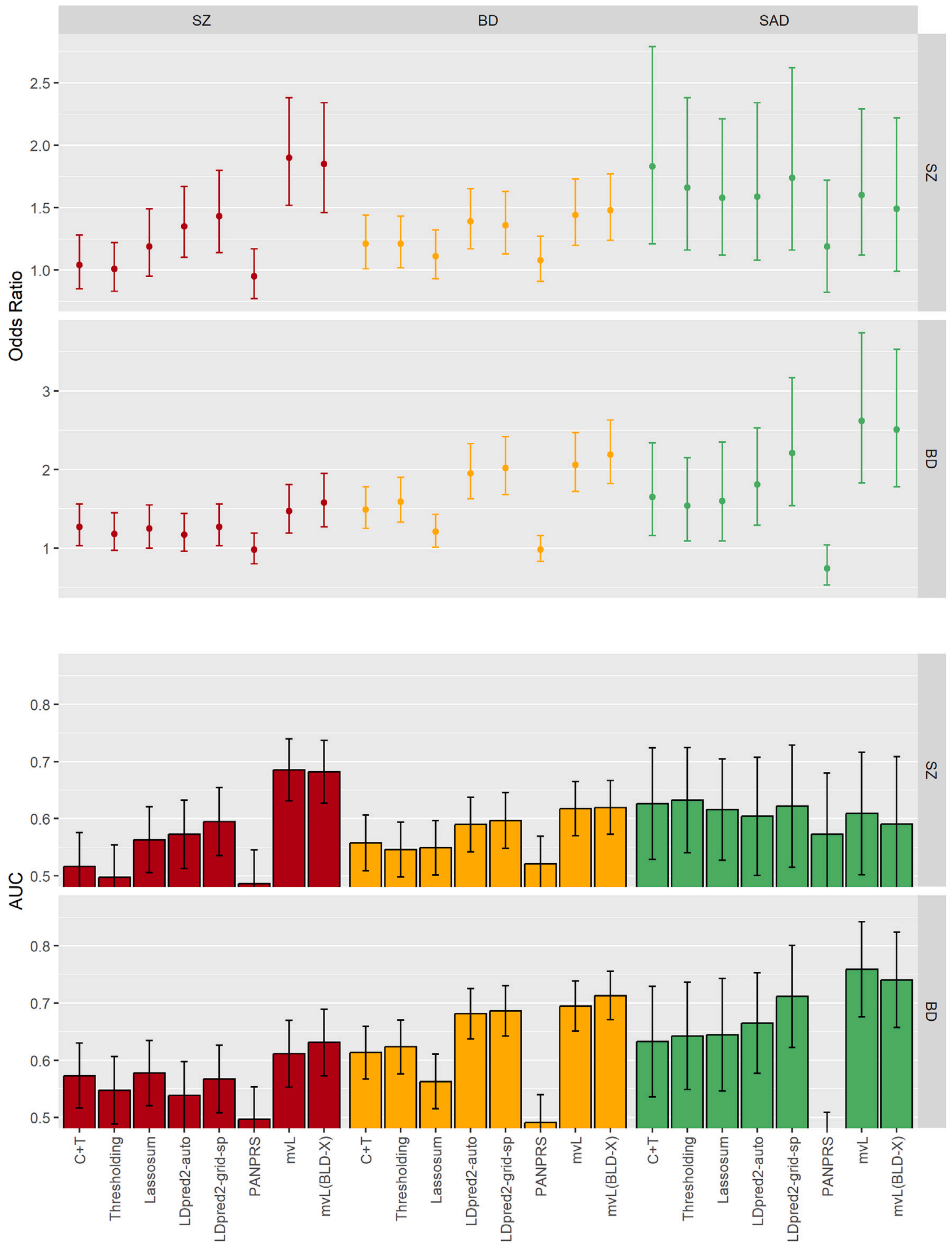

Figure 3. Predictive performance of Multivariate Lassosum for psychiatric traits in the Eastern Quebec schizophrenia and bipolar disorder study compared with clumping and thresholding, thresholding alone, Lassosum, PANPRS, and LDpred2

Top panel: odds ratio and 95% confidence interval of trait for an increase of 1 standard deviation in PRS; Bottom panel: area under the receiver operating curve (AUC) and 95% confidence interval for the prediction of the traits by PRS. mvL, Multivariate Lassosum with constant penalty and constant contribution of standardized genotypes of all SNPs; mvl(BLD-X), Multivariate Lassosum with constant penalty and baseline linkage disequilibrium model cross-trait; SZ, schizophrenia; BD, bipolar disorder; SAD, schizoaffective disorder.

Discussion

We have combined multivariate linear mixed models and a LASSO penalty to propose Multivariate Lassosum, a new summary-statistics-based multivariate penalized regression approach to the definition of PRS for genetically correlated traits. This multivariate analysis improved the prediction of each trait compared with analyzing either two traits jointly or each trait separately using alternative methods involving the selection of SNPs (PANPRS, thresholding with and without clumping, LDpred2, and the original Lassosum) on both summary statistics simulated under a variety of scenarios and actual SZ and BD summary statistics. LDpred2 and the original Lassosum were competitive with Multivariate Lassosum only with low trait polygenicity. There are other PRS definitions involving some form of shrinkage of the SNP coefficients without forcing them to 0, but none of them consistently beat LDpred2 in a thorough evaluation on actual data for psychiatric traits,[34] and we consider LDpred2 as representative of single-trait methods achieving top predictive performance.

We also adapted the S-LDXR method initially proposed to estimate the trans-ethnic genetic covariance of one trait to estimate the genetic covariance between two traits as a function of SNP annotations, and we found that using such a model (BLD-X) to predict SNP-specific contributions to the heritabilities and genetic covariance of two traits improved the predictive performance when simulated SNP effects actually depended on the included SNP annotations, even though the simulation model did not coincide with the analysis model (Figure 1). However, when the simulation model was very different from the BLD-X model, such as a mixture of four genetic covariance matrices, the predictive performance degraded, and then a constant genetic covariance matrix performed better than the BLD-X model (Figure S1). Thus, proximity of the analysis model to the true underlying model is important to maximize predictive performance, and the heritability model maximizing correlation between PRS and traits in a validation sample should be selected. When predicting SZ and BD in an actual sample of patients and non-affected relatives, the BLD-X model slightly improved predictive performance for a majority of the tested traits over the constant genetic covariance model, and otherwise the two models performed similarly. This is slightly more favorable to the BLD-X model than the results of Ni et al.,[34] who reported no advantage with a similar heritability model implemented in MegaPRS[1] for predicting SZ and major depressive disorder analyzed separately. Tissue-specific annotations can be added to the BLD-X model.[20] Whether adding brain-specific annotations improves the prediction of SZ and BD could be investigated in future work.

We focused our evaluation of the methods on a pair of traits, although the methodology and software code are general for $q \geq 2$ traits. This is essentially to limit computing time, which grows faster than linearly with the number of traits. Also, we set the total variance of each trait to 1. For quantitative traits, equal variance can be obtained by scaling the traits. For dichotomous traits, this equal variance assumption may not hold, and further investigation of the impact of different variances for the traits analyzed remains an important avenue of further research.

The covariance terms of the β added to the quadratic penalty in Equation 2 leads to BLUPs under a linear mixed model.[5] In the elastic net context of Multivariate Lassosum, a different penalty strength may be optimal, which could be implemented by an additional penalty parameter λ_1 to the term $\beta_j^\top \Sigma_{bj}^{-1} \beta_j$. Additional tuning parameters add to the computational burden but may not improve predictive performance significantly, as PANPRS exemplifies.

The improvements in prediction performance achieved by considering genetically correlated traits in the construction of PRS could be meaningful in conjunction with other factors. As with other complex traits, non-genetic factors play an important role in the etiology of SZ and BD, such as childhood trauma[36,37] and socio-economic factors.[38,39] Given the moderate proportions of variance of these psychiatric traits explained by PRSs, these other factors need to be considered together with PRS to further improve prediction, whether in help-seeking individuals[40] or in children at familial risk.[41] Genetic correlation among traits also complexifies the prediction of the specific disorder that an at-risk subject will express, as the same subject may exhibit high PRSs for multiple correlated traits. This may not be a serious impediment to implementing preventive interventions, as such interventions may be indicated to prevent several genetically correlated disorders, e.g., cognitive remediation therapy in the case of prevention of SZ and BD.

In conclusion, the availability of summary statistics for a multitude of genetically correlated traits offers an opportunity to improve risk prediction of complex disorders through PRS in conjunction with non-genetic factors. We have made available Multivariate Lassosum as a software package to implement prediction of genetically correlated traits under a penalized regression framework.

Data and code availability

Scripts in R code for the simulation study and analysis of schizophrenia and bipolar disorder are available on Github (https://github.com/abureau/multitrait_PRS_comparison). Simulated data to reproduce certain steps of the simulation study are available as Bahda, Meriem; Ricard, Jasmin; Bureau, Alexandre (2023), "multivariateLassosum_Simulations," Mendeley Data, v2: https://doi.org/10.17632/jxz9jwssf6.2. The data of the Eastern Quebec SZ and BD kindred study are available on request from the corresponding author. The data from the CARTaGENE project are available after approval of an access request submitted at https://www.cartagene.qc.ca/en/researchers/access-request.html. The data are not publicly available due to privacy and ethical restrictions.

Supplemental information

Supplemental information can be found online at https://doi.org/10.1016/j.xhgg.2023.100209.

Acknowledgments

We thank Osvaldo Espin-Garcia (University Health Network, Toronto) for providing us PANPRS64, an optimized version of the SummaryLasso package. We are grateful to Yvon C. Chagnon for managing our molecular biology laboratory, to the professional research assistants Louise Bélanger, Marie-Claude Boisvert, Linda René, Lisette Gagnon, Claudie Poirier, Nicole Leclerc, Julie Lamarche, Pierrette Boutin, Lise St-Germain, Mélanie Mercier, Jordie Croteau, Alain Fournier, Valérie Jomphe, and Isabel Moreau (Center de recherche CERVO), to Claudia Moreau (Université du Québec à Chicoutimi), and to the family members who participated in the Eastern Quebec SZ and BD kindred study, which was funded by a Canada Research Chair (#950-200810) in psychiatric genetics of which Maziade is the Chair and by Canadian Institutes of Health Research grants (#MOP-74430, #MOP-114988, #PCG-155471, and #PJT-175122). This research project was funded by the New Frontiers in Research Fund, Exploration grant 2019-00752. The data management system was supported by the Canada Foundation for Innovation Leadership Opportunity Fund (grant 27592).

Declaration of interests

The authors declare no competing interests.

Received: October 26, 2022
Accepted: May 17, 2023

Web resources

- Multivariate Lassosum: https://github.com/abureau/multivariateLassosum
- PANPRS: https://github.com/lsncibb/PANPRS
- Lassosum: https://github.com/tshmak/lassosum
- LDpred2: https://github.com/privefl/bigsnpr
- S-LDXR: https://github.com/huwenboshi/s-ldxr
- 1000 Genomes Project Phase 3 data: https://www.internationalgenome.org
- PGC summary statistics data: https://www.med.unc.edu/pgc/results-and-downloads
- Plink: https://www.cog-genomics.org/plink2/
- Shapeit2: https://mathgen.stats.ox.ac.uk/genetics_software/shapeit/shapeit.html
- Michigan Imputation Server: https://imputationserver.sph.umich.edu

References

1. Zhang, Q., Privé, F., Vilhjálmsson, B., and Speed, D. (2021). Improved genetic prediction of complex traits from individual-level data or summary statistics. Nat. Commun. *12*, 4192.
2. Khera, A.V., Chaffin, M., Aragam, K.G., Haas, M.E., Roselli, C., Choi, S.H., Natarajan, P., Lander, E.S., Lubitz, S.A., Ellinor, P.T., et al. (2018). Genome-wide polygenic scores for common diseases identify individuals with risk equivalent to monogenic mutations. Nat. Genet. *50*, 1219–1224.
3. Bulik-Sullivan, B., Finucane, H.K., Anttila, V., Gusev, A., Day, F.R., Loh, P.-R., Psychiatric Genomics Consortium; and Genetic Consortium for Anorexia Nervosa of the Wellcome Trust Case Control Consortium 3, and Duncan, L., et al. (2015). an atlas of genetic correlations across human diseases and traits. Nat. Genet. *47*, 1236–1241.
4. Maier, R., Moser, G., Chen, G.-B., Ripke, S., Cross-Disorder Working Group of the Psychiatric Genomics Consortium, Coryell, W., Potash, J.B., Scheftner, W.A., Shi, J., Weissman, M.M., et al. (2015). Joint analysis of psychiatric disorders increases accuracy of risk prediction for schizophrenia, bipolar disorder, and major depressive disorder. Am. J. Hum. Genet. *96*, 283–294.
5. Maier, R.M., Zhu, Z., Lee, S.H., Trzaskowski, M., Ruderfer, D.M., Stahl, E.A., Ripke, S., Wray, N.R., Yang, J., Visscher, P.M., and Robinson, M.R. (2018). Improving genetic prediction by leveraging genetic correlations among human diseases and traits. Nat. Commun. *9*, 989.
6. Hu, Y., Lu, Q., Liu, W., Zhang, Y., Li, M., and Zhao, H. (2017). Joint modeling of genetically correlated diseases and functional annotations increases accuracy of polygenic risk prediction. PLoS Genet. *13*, e1006836.
7. Mak, T.S.H., Porsch, R.M., Choi, S.W., Zhou, X., and Sham, P.C. (2017). Polygenic scores via penalized regression on summary statistics. Genet. Epidemiol. *41*, 469–480.
8. Chen, T.-H., Chatterjee, N., Landi, M.T., and Shi, J. (2021). A penalized regression framework for building polygenic risk models based on summary statistics from genome-wide association studies and incorporating external information. J. Am. Stat. Assoc. *116*, 133–143.
9. Speed, D., Holmes, J., and Balding, D.J. (2020). Evaluating and improving heritability models using summary statistics. Nat. Genet. *52*, 458–462.
10. Privé, F., Arbel, J., and Vilhjálmsson, B.J. (2020). LDpred2: better, faster, stronger. Bioinformatics *36*, 5424–5431.
11. International Schizophrenia Consortium, Purcell, S.M., Wray, N.R., Stone, J.L., Visscher, P.M., O'Donovan, M.C., Sullivan, P.F., and Sklar, P. (2009). Common polygenic variation contributes to risk of schizophrenia and bipolar disorder. Nature *460*, 748–752.
12. Jiang, J. (2007). Linear and generalized linear mixed models and their applications. In Springer Series in Statistics (Springer New York).
13. Tibshirani, R. (1996). Regression shrinkage and selection via the Lasso. J. Roy. Stat. Soc. B *58*, 267–288.
14. Zou, H. (2006). The adaptive lasso and its oracle properties. J. Am. Stat. Assoc. *101*, 1418–1429.
15. Speed, D., and Balding, D.J. (2019). SumHer better estimates the SNP heritability of complex traits from summary statistics. Nat. Genet. *51*, 277–284.
16. Zou, H., and Hastie, T. (2005). Regularization and variable selection via the elastic net. J. R. Stat. Soc. Ser. B-Stat. Methodol. *67*, 301–320.
17. Bühlmann, P., and Geer, S.A.v. d. (2011). Statistics for high-dimensional data: methods, theory and applications. In Springer Series in Statistics (Springer, Heidelberg).
18. Mak, T.S.H., Kwan, J.S.H., Campbell, D.D., and Sham, P.C. (2016). Local true Discovery rate weighted polygenic scores using GWAS summary data. Behav. Genet. *46*, 573–582.

19. Gazal, S., Finucane, H.K., Furlotte, N.A., Loh, P.-R., Palamara, P.F., Liu, X., Schoech, A., Bulik-Sullivan, B., Neale, B.M., Gusev, A., and Price, A.L. (2017). Linkage disequilibrium-dependent architecture of human complex traits shows action of negative selection. Nat. Genet. *49*, 1421–1427.
20. Shi, H., Gazal, S., Kanai, M., Koch, E.M., Schoech, A.P., Siewert, K.M., Kim, S.S., Luo, Y., Amariuta, T., Huang, H., et al. (2021). Population-specific causal disease effect sizes in functionally important regions impacted by selection. Nat. Commun. *12*, 1098.
21. Awadalla, P., Boileau, C., Payette, Y., Idaghdour, Y., Goulet, J.-P., Knoppers, B., Hamet, P., Laberge, C.; and CARTaGENE Project (2013). Cohort profile of the cartagene study: quebec's population-based biobank for public health and personalized genomics. Int. J. Epidemiol. *42*, 1285–1299.
22. Trubetskoy, V., Pardiñas, A.F., Qi, T., Panagiotaropoulou, G., Awasthi, S., Bigdeli, T.B., Bryois, J., Chen, C.-Y., Dennison, C.A., Hall, L.S., et al. (2022). Mapping genomic loci implicates genes and synaptic biology in schizophrenia. Nature *604*, 502–508.
23. Mullins, N., Forstner, A.J., O'Connell, K.S., Coombes, B., Coleman, J.R.I., Qiao, Z., Als, T.D., Bigdeli, T.B., Børte, S., Bryois, J., et al. (2021). Genome-wide association study of more than 40,000 bipolar disorder cases provides new insights into the underlying biology. Nat. Genet. *53*, 817–829.
24. Frei, O., Holland, D., Smeland, O.B., Shadrin, A.A., Fan, C.C., Maeland, S., O'Connell, K.S., Wang, Y., Djurovic, S., Thompson, W.K., et al. (2019). Bivariate causal mixture model quantifies polygenic overlap between complex traits beyond genetic correlation. Nat. Commun. *10*, 2417.
25. Turley, P., Walters, R.K., Maghzian, O., Okbay, A., Lee, J.J., Fontana, M.A., Nguyen-Viet, T.A., Wedow, R., Zacher, M., Furlotte, N.A., et al. (2018). Multi-trait analysis of genome-wide association summary statistics using mtag. Nat. Genet. *50*, 229–237.
26. Van Snellenberg, J.X., and de Candia, T. (2009). Meta-analytic evidence for familial coaggregation of schizophrenia and bipolar disorder. Arch. Gen. Psychiatry *66*, 748–755.
27. Maziade, M., Roy, M.-A., Chagnon, Y.C., Cliche, D., Fournier, J.-P., Montgrain, N., Dion, C., Lavallée, J.C., Garneau, Y., Gingras, N., et al. (2005). Shared and specific susceptibility loci for schizophrenia and bipolar disorder: a dense genome scan in eastern quebec families. Mol. Psychiatry *10*, 486–499.
28. Chagnon, Y.C., Maziade, M., Paccalet, T., Croteau, J., Fournier, A., Roy, M.-A., and Bureau, A. (2020). A multimodal attempt to follow-up linkage regions using RNA expression, SNPs and CpG methylation in schizophrenia and bipolar disorder kindreds. Eur. J. Hum. Genet. *28*, 499–507.
29. Maziade, M., Roy, M.A., Fournier, J.P., Cliche, D., Mérette, C., Caron, C., Garneau, Y., Montgrain, N., Shriqui, C., and Dion, C. (1992). Reliability of best-estimate diagnosis in genetic linkage studies of major psychoses: results from the quebec pedigree studies. Am. J. Psychiatr. *149*, 1674–1686.
30. Roy, M.A., Lanctôt, G., Mérette, C., Cliche, D., Fournier, J.P., Boutin, P., Rodrigue, C., Charron, L., Turgeon, M., Hamel, M., et al. (1997). Clinical and methodological factors related to reliability of the best-estimate diagnostic procedure. Am. J. Psychiatr. *154*, 1726–1733.
31. Boies, S., Mérette, C., Paccalet, T., Maziade, M., and Bureau, A. (2018). Polygenic risk scores distinguish patients from non-affected adult relatives and from normal controls in schizophrenia and bipolar disorder multi-affected kindreds. Am. J. Med. Genet. B Neuropsychiatr. Genet. *177*, 329–336.
32. O'Connell, J., Gurdasani, D., Delaneau, O., Pirastu, N., Ulivi, S., Cocca, M., Traglia, M., Huang, J., Huffman, J.E., Rudan, I., et al. (2014). A general approach for haplotype phasing across the full spectrum of relatedness. PLoS Genet. *10*, e1004234.
33. McCarthy, S., Das, S., Kretzschmar, W., Delaneau, O., Wood, A.R., Teumer, A., Kang, H.M., Fuchsberger, C., Danecek, P., Sharp, K., et al. (2016). A reference panel of 64,976 haplotypes for genotype imputation. Nat. Genet. *48*, 1279–1283.
34. Ni, G., Zeng, J., Revez, J.A., Wang, Y., Zheng, Z., Ge, T., Restuadi, R., Kiewa, J., Nyholt, D.R., Coleman, J.R.I., et al. (2021). A comparison of ten polygenic score methods for psychiatric disorders applied across multiple cohorts. Biol. Psychiatr. *90*, 611–620.
35. Chang, C.C., Chow, C.C., Tellier, L.C., Vattikuti, S., Purcell, S.M., and Lee, J.J. (2015). Second-generation plink: rising to the challenge of larger and richer datasets. GigaScience *4*, 7.
36. Berthelot N., Garon-Bissonnette J., Jomphe V., Doucet-Beaupré H., Bureau A., Maziade M. Childhood trauma may increase risk of psychosis and mood disorder in genetically high-risk children and Adolescents by enhancing the Accumulation of risk indicators. Schizophrenia Bulletin Open 2022;3,sgac017.
37. Kelleher, I., Keeley, H., Corcoran, P., Ramsay, H., Wasserman, C., Carli, V., Sarchiapone, M., Hoven, C., Wasserman, D., and Cannon, M. (2013). Childhood trauma and psychosis in a prospective cohort study: cause, effect, and directionality. Am. J. Psychiatr. *170*, 734–741.
38. Sareen, J., Afifi, T.O., McMillan, K.A., and Asmundson, G.J.G. (2011). Relationship between household income and mental disorders: findings from a population-based longitudinal study. Arch. Gen. Psychiatry *68*, 419–427.
39. Silva, M., Loureiro, A., and Cardoso, G. (2016). Social determinants of mental health: a review of the evidence. Eur. J. Psychiatr. *30*, 259–292.
40. Murray, G.K., Lin, T., Austin, J., McGrath, J.J., Hickie, I.B., and Wray, N.R. (2021). Could polygenic risk scores Be useful in psychiatry?: a review. JAMA Psychiatr. *78*, 210–219.
41. Maziade, M. (2017). At risk for serious mental illness — screening children of patients with mood disorders or schizophrenia. N. Engl. J. Med. *376*, 910–912.

HGGA, Volume *4*

Supplemental information

Multivariate extension of penalized regression on summary statistics to construct polygenic risk scores for correlated traits

Meriem Bahda, Jasmin Ricard, Simon L. Girard, Michel Maziade, Maripier Isabelle, and Alexandre Bureau

Supplemental information

1 Simulation parameters

For the mixture-of-four-genetic-covariance-matrices scenario, we generated the vector (β_{j1}, β_{j2}) of genetic effects for the SNP j for the trait 1 and trait 2 as follows :

$$\begin{pmatrix} \beta_{j1} \\ \beta_{j2} \end{pmatrix} \sim \begin{cases} N\left(\begin{bmatrix} 0 \\ 0 \end{bmatrix}, \begin{bmatrix} \sigma^2 & \sigma^2 r \\ \sigma^2 r & \sigma^2 \end{bmatrix}\right) & \text{with probability } \pi_1 \quad (C=1) \\ \begin{bmatrix} N\left(0, \sigma^2\right) \\ 0 \end{bmatrix} & \text{with probability } \pi_2 \quad (C=2) \\ \begin{bmatrix} 0 \\ N\left(0, \sigma^2\right) \end{bmatrix} & \text{with probability } \pi_3 \quad (C=3) \\ \begin{bmatrix} 0 \\ 0 \end{bmatrix} & \text{with probability } \pi_4 \quad (C=4) \end{cases} \tag{1}$$

with

$$\begin{aligned} &\cdot \operatorname{Cov}\left(\beta_{j1}, \beta_{j2}\right) = \operatorname{Cov}\left(\beta_{j1}, \beta_{j2} \mid C=1\right) \cdot P(C=1) = \sigma^2 r \pi_1, \\ &\cdot \operatorname{Var}\left(\beta_{j1}\right) = \operatorname{Var}\left(\beta_{j1} \mid C \leqslant 2\right) \cdot P(C \leqslant 2) = \sigma^2\left(\pi_1+\pi_2\right), \\ &\cdot \operatorname{Var}\left(\beta_{j2}\right) = \operatorname{Var}\left(\beta_{j2} \mid C \in \{1,3\}\right) \cdot P(C \in \{1,3\}) = \sigma^2\left(\pi_1+\pi_3\right), \\ &\cdot \pi_1+\pi_2+\pi_3+\pi_4 = 1. \end{aligned} \tag{2}$$

We have 6 unknown parameters, $\pi_1, \pi_1, \pi_3, \pi_4, \sigma, r$, and 4 equations. We chose to specify the value of the parameters π_1 and σ in order to solve the equations.

It is important to note that we have the following constraints when we choose the value of π_1 :

$$\begin{aligned} \pi_1 &> \frac{\operatorname{Cov}\left(\beta_{j1}, \beta_{j2}\right)}{\sigma^2}, \\ \pi_1 &< min\left(\frac{\operatorname{Var}\left(\beta_{j1}\right)}{\sigma^2}, \frac{\operatorname{Var}\left(\beta_{j2}\right)}{\sigma^2}\right). \end{aligned}$$

Otherwise, we obtain negative values for the parameters π_2 and π_3, and a value of r greater than 1. We note that under the constant heritability and genetic covariance scenario, $\operatorname{Cov}\left(\beta_{j1}, \beta_{j2}\right) = \frac{1}{p}\rho_g$ and $\operatorname{Var}\left(\beta_{jk}\right) = \frac{1}{p}h_{gk}^2, k = 1, 2$, i.e. they are proportional to the prespecified heritabilities and genetic covariance.

The value of σ^2 was chosen according to the number of SNPs used in the simulations, in order to obtain targeted ranges of values for π_1. For the high polygenicity scenario, we set $\sigma^2 = 2.00e-6$ with 479,158 SNPs and $\sigma^2 = 2.26e-6$ with 423,552 SNPs in order to have a π_1 that varies within the interval [0.29 ; 0.45]. We then set $\pi_1 = 0.35$ and obtained $\pi_2 = 0.15$, $\pi_3 = 0.13$, $\pi_4 = 0.37$ and $r = 0.79$ by solving the system of equations (2). For the low polygenicity scenario, we set $\sigma^2 = 1.06e-5$ and $\pi_1 = 0.08$ and obtained $\pi_2 = 0.024$, $\pi_3 = 0.02$, $\pi_4 = 0.876$ and $r = 0.88$ by solving the system of equations (2). Table S1 presents the corresponding number of SNPs for each scenario.

For the scenario where the heritabilities and genetic covariance of the two traits depend on predictions from BLD-X model annotations we kept the same πs. Given the predicted contribution to heritability of either trait was negative for a number of SNPs and was thus set to 0 to begin with, the selection of causal SNPs for the simulation was restricted. We present below the steps for the simulations with 423,552 SNPs and high polygenicity. Similar steps were followed for 479,158 SNPs or low polygenicity.

1. The 148,243 SNPs causal for both traits (35% of 423,552) were selected among the 282,194 SNPs with $\hat{h}^2_{g1}(j) > 0$ and $\hat{h}^2_{g2}(j) > 0$.
2. The 59,445 SNPs causal for trait 1 only were selected among the SNPs remaining from step 1 and the 37,293 SNPs with $\hat{h}^2_{g1}(j) > 0$ and $\hat{h}^2_{g2}(j) \leq 0$.
3. The 50,607 SNPs causal for trait 2 only were selected among the SNPs remaining from step 1 not selected at step 2 and the 45,184 SNPs with $\hat{h}^2_{g1}(j) \leq 0$ and $\hat{h}^2_{g2}(j) > 0$.
4. The effect of all remaining SNPs was set to 0.

The non-zero SNP effects were then simulated according to equation (7) from the main text.

2 Genotyping quality control procedures

SNPs with the following quality problems were removed: missing call rate higher than 0.02, SNP mismatches with Haplotype reference consortium (HRC), SNPs which are indels, minor allele frequency difference with HRC > 0.2, palindromic SNP with frequency > 0.4, allele mismatch with HRC, duplicates, Hardy Weinberg equilibrium test p-value lower than 10^{-7}. From the first wave of SNP array genotyping using an Illumina Omni Express chips at Genome Québec, a total of 507 subjects were genotyped at 656,032 autosomal SNPs. A total of 33,848 SNPs were removed following quality control. Imputation using the Michigan Imputation Server resulted in a total of 39,127,678 imputed SNPs.

From the second wave of SNP array genotyping using an Illumina Global Screening Array at Genome Québec, a total of 615 subjects were genotyped at 691,719 autosomal SNPs. Two subjects were removed: one, thought to be a brother of three other subjects, turned out to be an half-brother after analysis with Prest-plus software and the other presented a sex disconcordance along with a high rate of Mendelian errors. A total of 188,854 SNPs were removed following quality control. Imputation using the Michigan Imputation Server resulted in a total of 39,131,578 imputed SNPs.

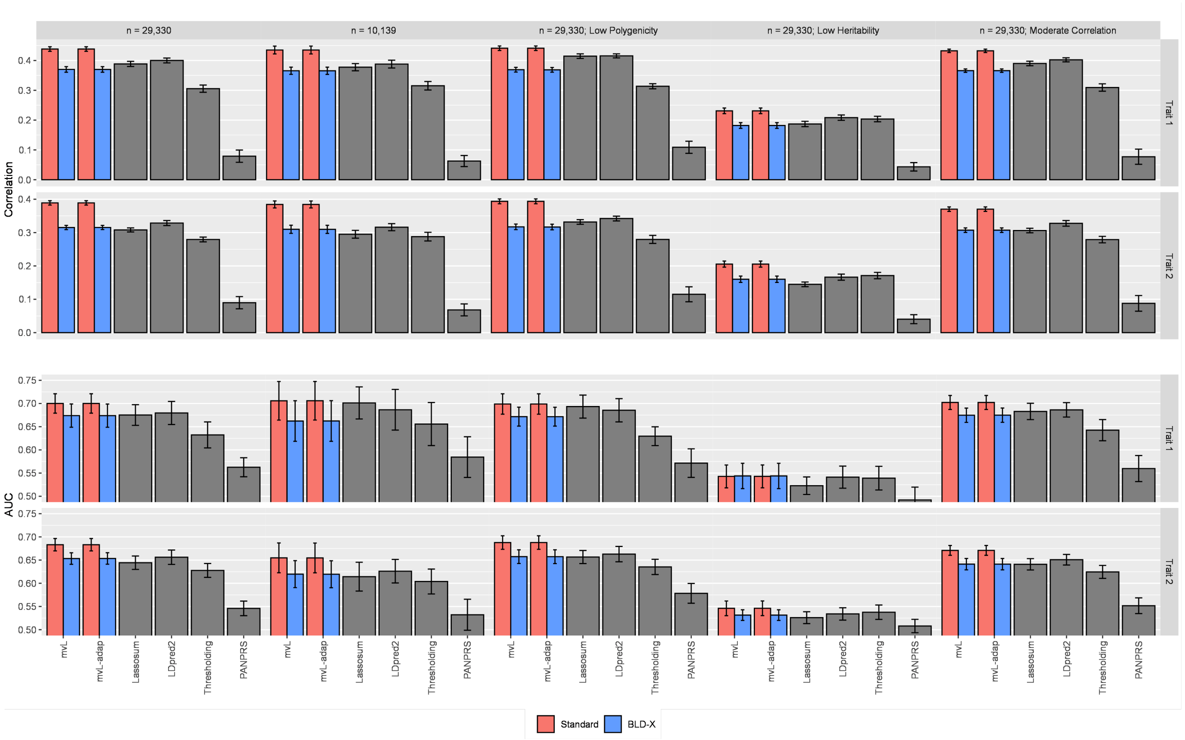

Figure S1: Comparison of PRS predictive performance for multiple variations of the simulation scenario with a mixture of four constant genetic covariance matrices. Top panel: Pearson correlation of the PRS with the true predictor. Bottom panel: area under the receiver operating curve (AUC) for the prediction of simulated traits by PRS. Mean and 95% confidence interval based on 20 replicates. The penalty parameter λ (for the penalized regression methods) and the threshold for thresholding were set to the values maximizing the correlation between the PRS and the trait y in a validation set. Models for heritability and covariance used in analysis : BLD-X: Baseline linkage disequilibrium model-cross trait; Standard: constant contribution of standardized genotypes of all SNPs.

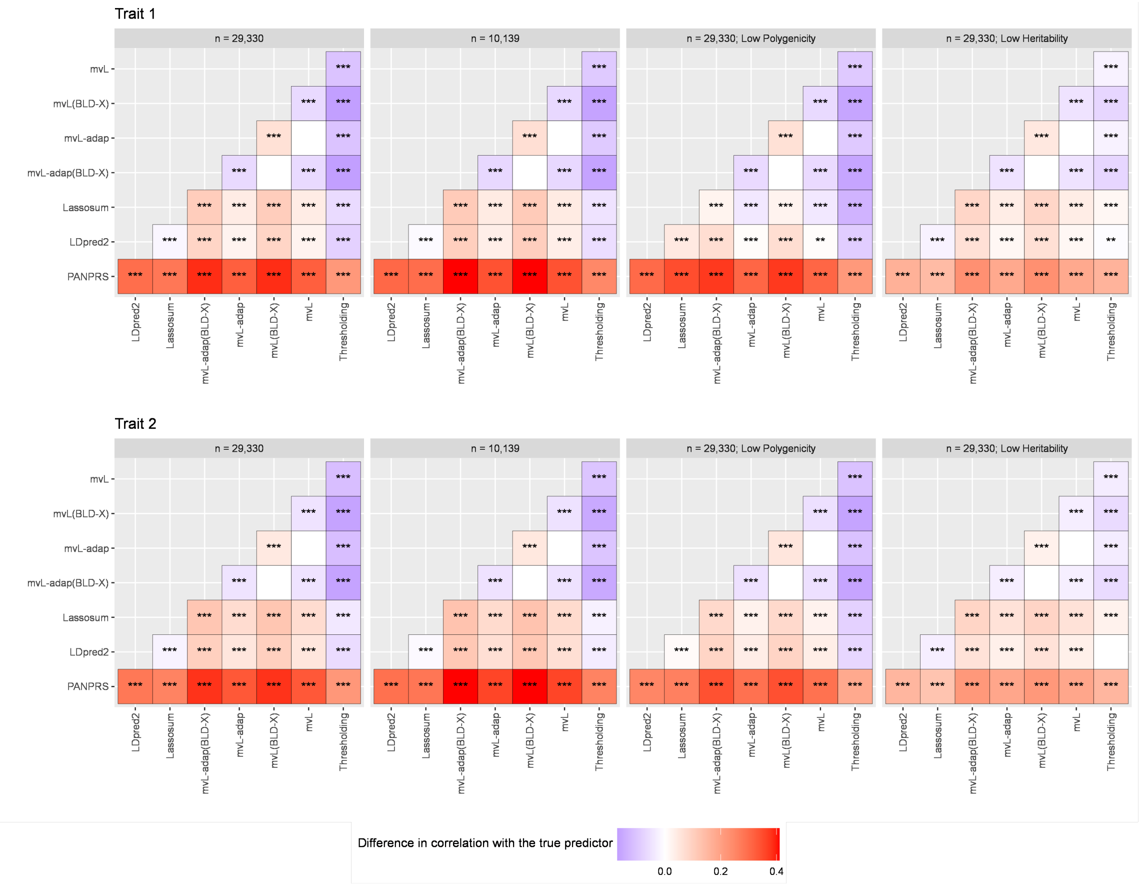

Figure S2: Difference in Pearson correlation of the PRS with the true predictor between methods under various simulation scenarios with heritability and genetic covariance of the two traits depending on genomic annotations. See legend of Figure 1 for the definitions of acronyms for the methods. Statistical significance of the difference evaluated by paired t-tests using 20 replicates, and p-values were corrected for multiple testing using the Bonferroni method. *: $p < 0.05$, **: $p < 5 \times 10^{-4}$, ***: $p < 5 \times 10^{-8}$

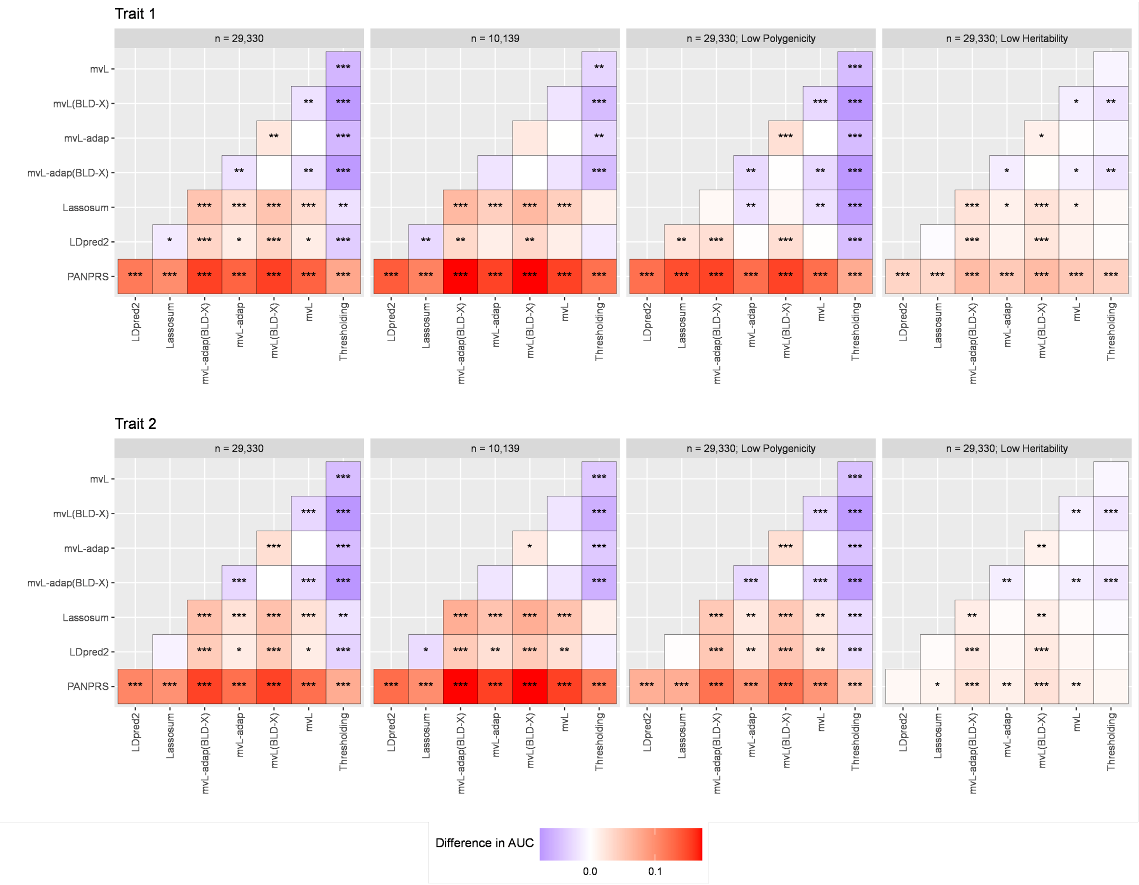

Figure S3: Difference in area under the receiver operating curve (AUC) for the prediction of simulated traits by PRS under various simulation scenarios with heritability and genetic covariance of the two traits depending on genomic annotations. See legend of Figure 1 for the definitions of acronyms for the methods. Statistical significance of the difference evaluated by paired t-tests using 20 replicates, and p-values were corrected for multiple testing using the Bonferroni method. *: $p < 0.05$, **: $p < 5 \times 10^{-4}$, ***: $p < 5 \times 10^{-8}$

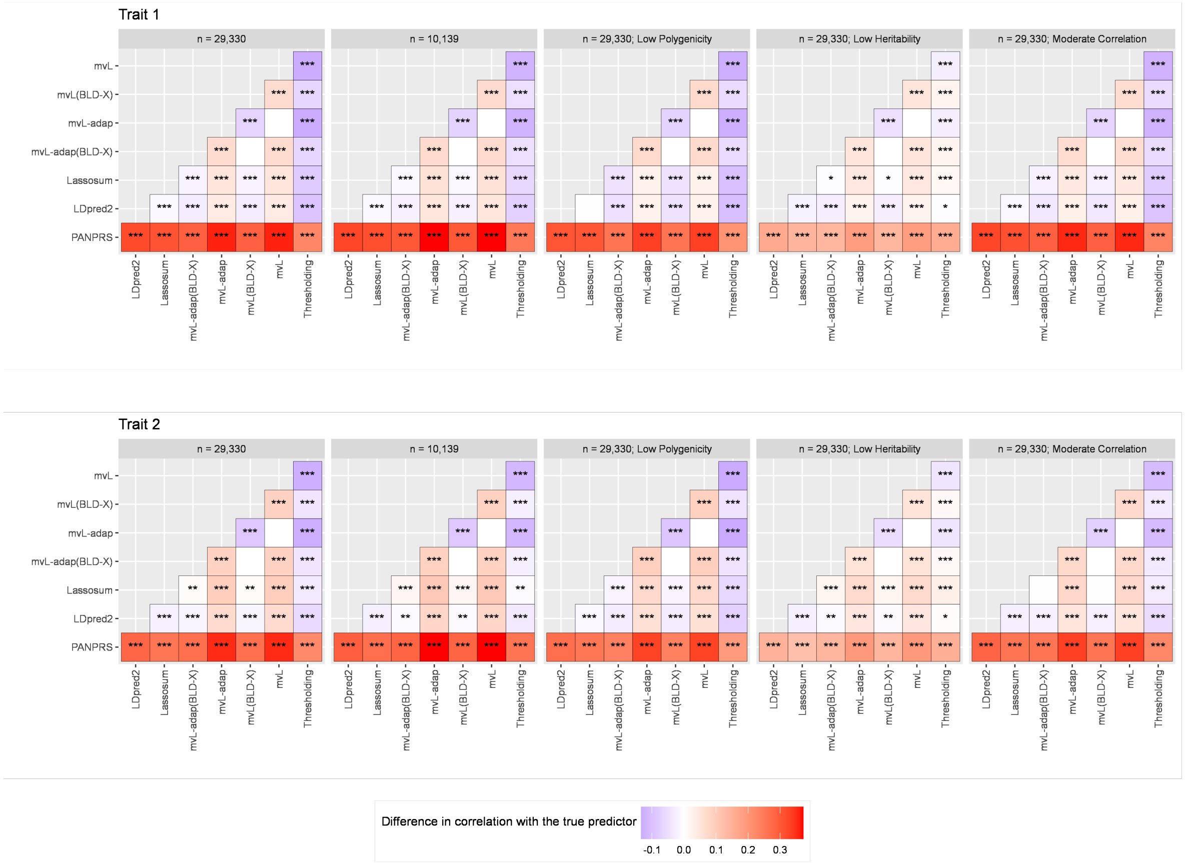

Figure S4: Difference in Pearson correlation of the PRS with the true predictor between methods under various simulation scenarios with a mixture of four constant genetic covariance matrices. See legend of Figure 1 for the definitions of acronyms for the methods. Statistical significance of the difference evaluated by paired t-tests using 20 replicates, and p-values were corrected for multiple testing using the Bonferroni method. *: $p < 0.05$, **: $p < 5 \times 10^{-4}$, ***: $p < 5 \times 10^{-8}$

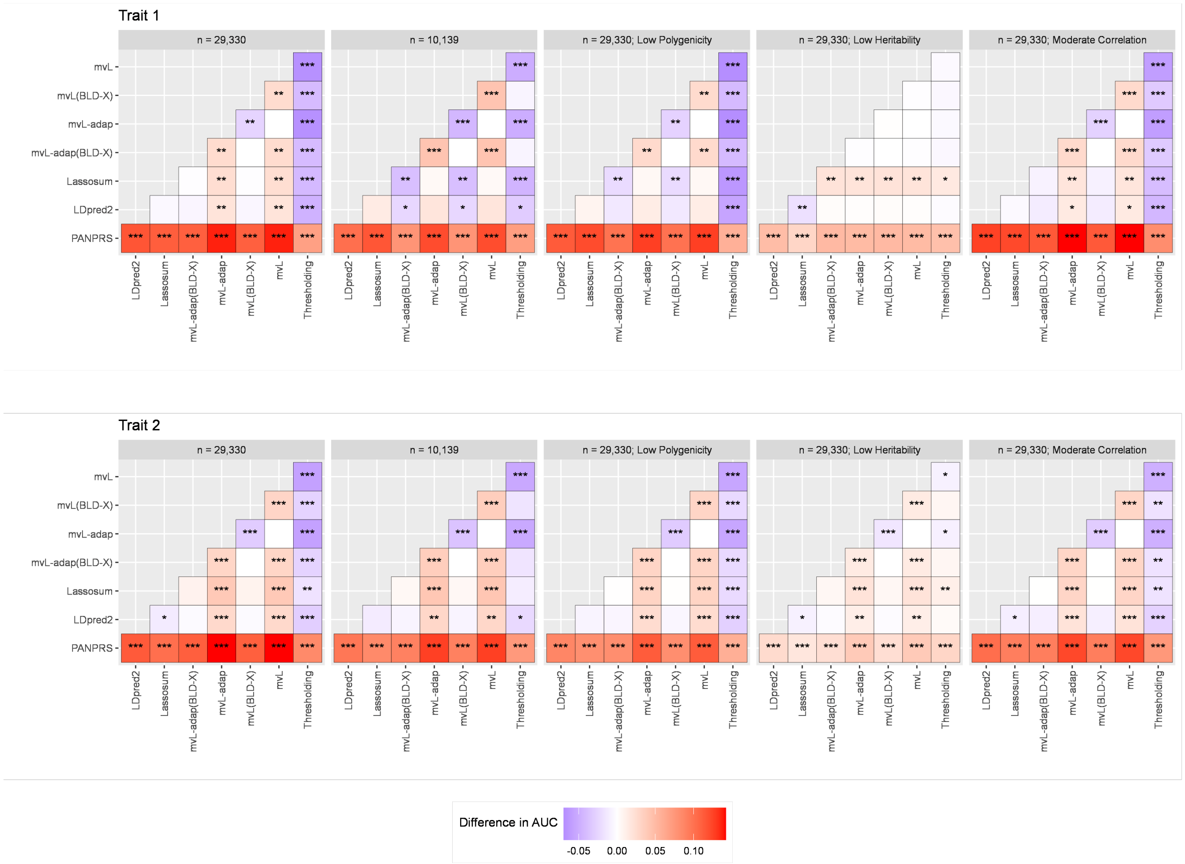

Figure S5: Difference in area under the receiver operating curve (AUC) for the prediction of simulated traits by PRS under various simulation scenarios with a mixture of four constant genetic covariance matrices. See legend of Figure 1 for the definitions of acronyms for the methods. Statistical significance of the difference evaluated by paired t-tests using 20 replicates, and p-values were corrected for multiple testing using the Bonferroni method. *: $p < 0.05$, **: $p < 5 \times 10^{-4}$, ***: $p < 5 \times 10^{-8}$

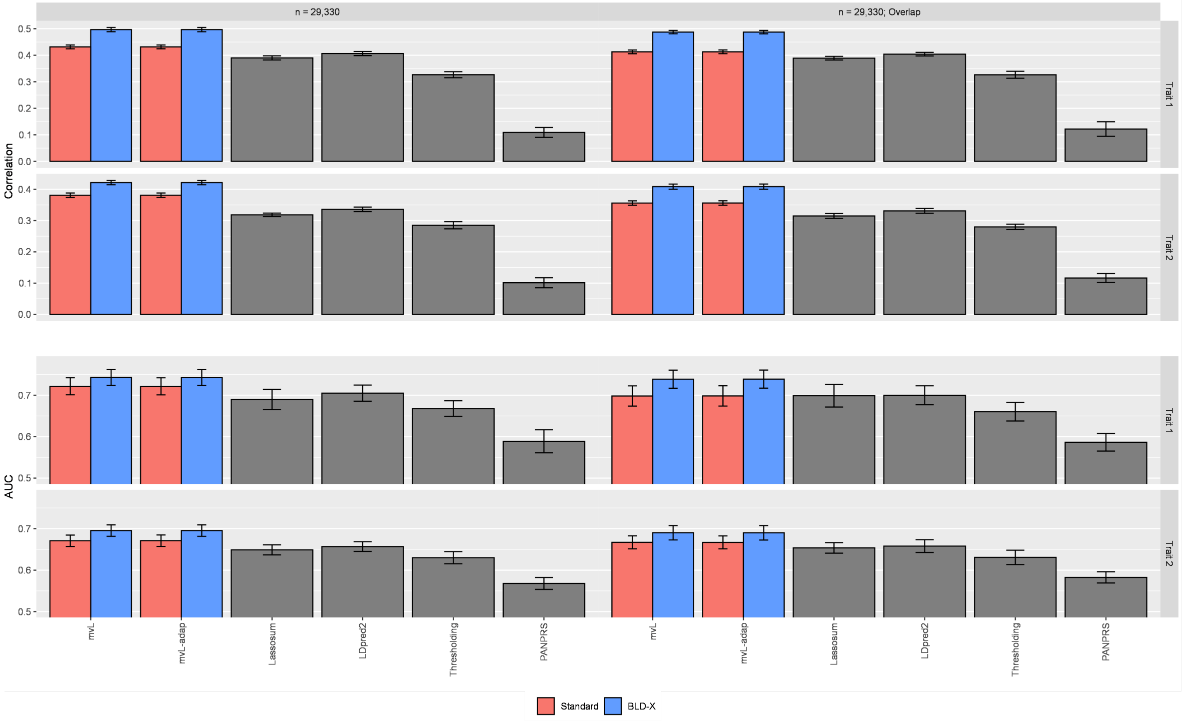

Figure S6: Comparison of PRS predictive performance in presence of simulated sample overlap with between-trait summary-statistics correlation $\hat{\rho}_o = 0.32$ compared to independent samples under the simulation scenario where heritability and genetic covariance of two traits depend on genomic annotations. Top panel: Pearson correlation of the PRS with the true predictor. Bottom panel: area under the receiver operating curve (AUC) for the prediction of simulated traits by PRS. Mean and 95% confidence interval based on 20 replicates. The penalty parameter λ (for the penalized regression methods) and the threshold for thresholding were set to the values maximizing the correlation between the PRS and the trait y in a validation set. Methods compared: mvL: Multivariate Lassosum with constant penalty, mvL-adapt: Multivariate Lassosum with adaptive penalty based on the initial estimates from mvL. Models for heritability and covariance used in analysis : BLD-X: Baseline linkage disequilibrium model-cross trait; Standard: constant contribution of standardized genotypes of all SNPs.

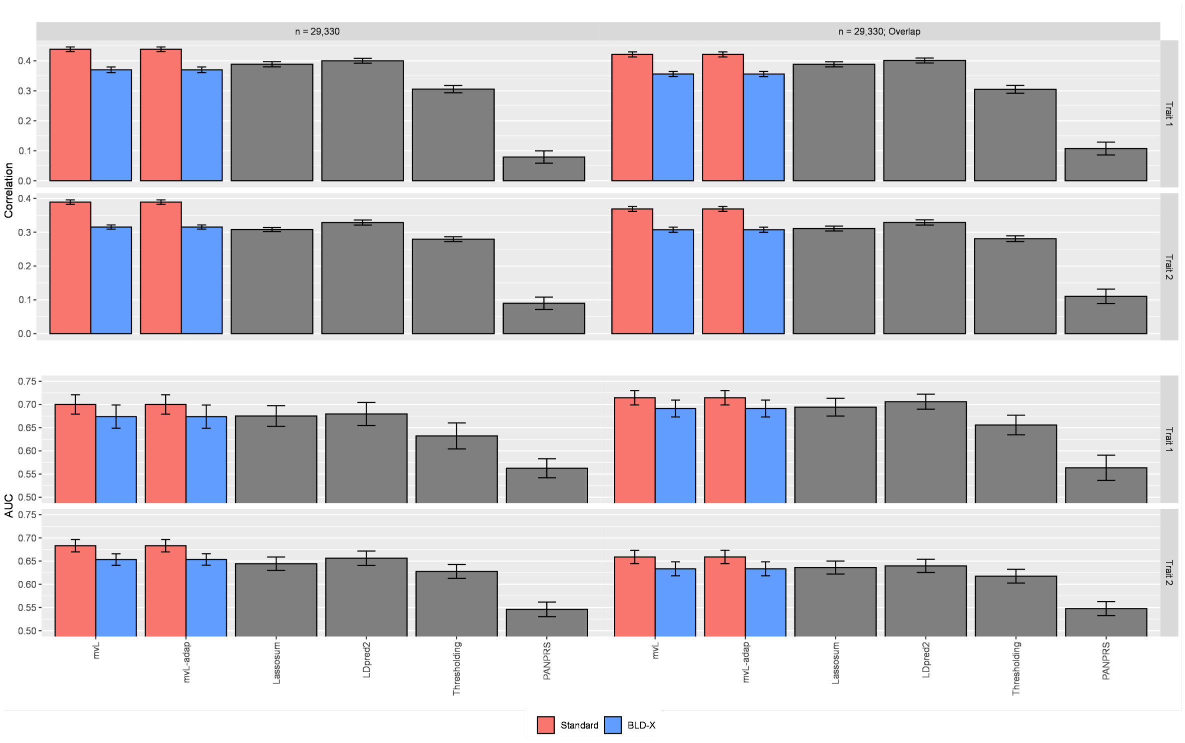

Figure S7: Comparison of PRS predictive performance in presence of simulated sample overlap with between-trait summary-statistics correlation $\hat{\rho}_o = 0.32$ compared to independent samples under the simulation scenario with a mixture of four constant genetic covariance matrices. Top panel: Pearson correlation of the PRS with the true predictor. Bottom panel: area under the receiver operating curve (AUC) for the prediction of simulated traits by PRS. Mean and 95% confidence interval based on 20 replicates. The penalty parameter λ (for the penalized regression methods) and the threshold for thresholding were set to the values maximizing the correlation between the PRS and the trait y in a validation set. Methods compared: mvL: Multivariate Lassosum with constant penalty, mvL-adapt: Multivariate Lassosum with adaptive penalty based on the initial estimates from mvL. Models for heritability and covariance used in analysis : BLD-X: Baseline linkage disequilibrium model-cross trait; Standard: constant contribution of standardized genotypes of all SNPs.

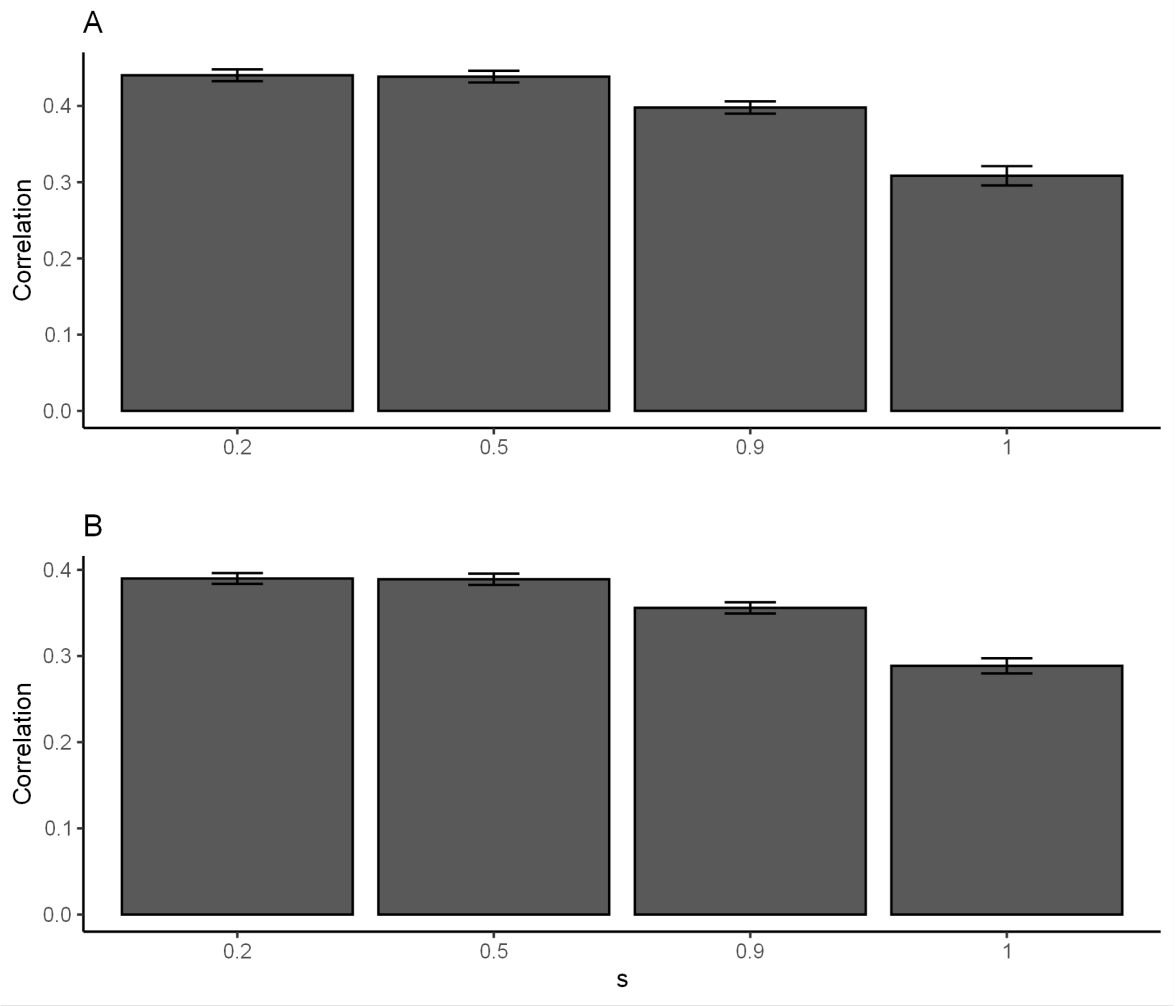

Figure S8: Pearson correlation of the PRS with the true predictor as a function of the s regularization parameter under the reference simulation scenario. A) Heritability and genetic covariance of two traits depending on genomic annotations B) Mixture of four constant genetic covariance matrices.

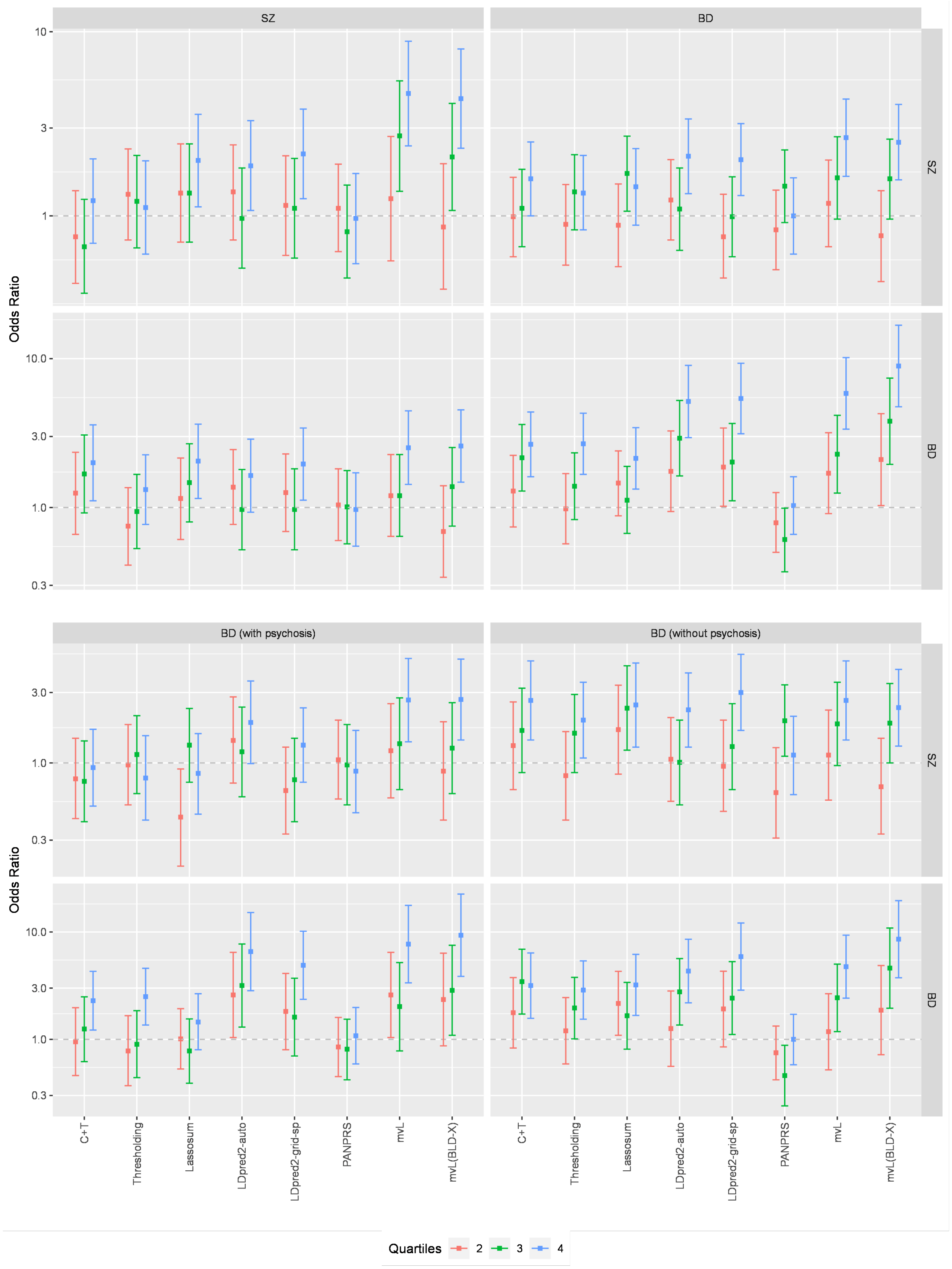

Figure S9: Odds ratios of psychiatric diagnoses for the second to fourth quartiles of PRS defined in the non-affected adult relatives compared to the first quartile based on various PRS definitions. Estimates and 95% confidence intervals. Each column is a predicted trait and each row a trait on which summary statistics were obtained to construct PRSs. SZ : schizophrenia, BD: Bipolar disorder. Schizoaffective disorder was excluded due to the small number of cases.

Table S1: Number of causal SNPs for each trait combination under different numbers of SNPs and polygenicity levels

Traits	High Polygenicity			Low Polygenicity	
	π^a	n = 10,139<br>p = 479,158	n = 29,330<br>p = 423,552	π^a	n = 29,330<br>p = 423,552
1 and 2	0.35	167,705	148,243	0.080	33,884
1	0.14	67,082	59,445	0.024	10,353
2	0.12	57,499	50,607	0.020	8,471
None	0.39	186,872	165,257	0.876	370,844

[a]: Proportion of causal SNPs in each case

Table S2: Features of the polygenic risk score methods

Method	Parameters	Use of validation sample	Heritability model	Clumping (in real data)
C + T[a]	P-value threshold,<br>Correlation threshold	Yes	NA	Yes
Thresholding	P-value threshold	Yes	NA	No
Lassosum	λ: LASSO penalty,<br>s: R regularization	Yes	Equal	No
LDpred2-auto	p: Proportion of causal variants,<br>h^2: Heritability	No	Equal	No
LDpred2-grid-sp	p: Proportion of causal variants<br>h^2: Heritability	Yes	Equal	No
PANPRS	λ_0: LASSO penalty,<br>λ_1: Traits total effect penalty	Yes	Equal	Yes
mvL[b]	λ: LASSO penalty,<br>s: R regularization	Yes	Equal or BLD-X[c]	No

[a]: Clumping and thresholding
[b]: Multivariate Lassosum
[c]: Baseline linkage disequilibrium model-cross trait

Table S3: Predictive performance of PRSs on psychiatric traits in the Eastern Quebec schizophrenia and bipolar disorder study

SS[a]	Trait	n	Method	OR (95% CI)[b]	P-value	R^{2c}	$R^{2c}_{\text{liab.}}$	AUC
SZ[h]	SZ	Cases = 124 NAARs[d] = 442	C+T[e]	1.04 (0.85-1.28)	0.679	3×10^{-4}	3×10^{-4}	0.516
			Thresholding	1.01 (0.83-1.22)	0.960	0.000	0.000	0.497
			Lassosum	1.19 (0.95-1.49)	0.126	0.006	0.004	0.563
			LDpred2-auto	1.35 (1.10-1.67)	0.005	0.018	0.012	0.573
			LDpred2-grid-sp	1.43 (1.14-1.80)	0.002	0.025	0.016	0.595
			PANPRS	0.95 (0.77-1.17)	0.649	5×10^{-4}	3×10^{-4}	0.486
			mvL[f]	1.90 (1.52-2.38)	2×10^{-8}	0.079	0.051	0.685
			mvL(BLD-X[g])	1.85 (1.46-2.34)	3×10^{-7}	0.074	0.047	0.682
	BD	Cases = 205 NAARs = 442	C+T	1.21 (1.01-1.44)	0.041	0.008	0.005	0.558
			Thresholding	1.21 (1.02-1.43)	0.025	0.008	0.006	0.546
			Lassosum	1.11 (0.93-1.32)	0.261	0.003	0.002	0.549
			LDpred2-auto	1.39 (1.17-1.65)	2×10^{-4}	0.023	0.016	0.590
			LDpred2-grid-sp	1.36 (1.13-1.63)	9×10^{-4}	0.022	0.015	0.596
			PANPRS	1.08 (0.91-1.27)	0.381	0.001	9×10^{-4}	0.522
			mvL	1.44 (1.20-1.73)	7×10^{-5}	0.032	0.021	0.618
			mvL(BLD-X)	1.48 (1.24-1.77)	2×10^{-5}	0.035	0.024	0.620
	SAD	Cases = 35 NAARs = 442	C+T	1.83 (1.21-2.79)	0.005	0.019	0.025	0.626
			Thresholding	1.66 (1.16-2.38)	0.006	0.015	0.023	0.633
			Lassosum	1.58 (1.12-2.21)	0.008	0.009	0.016	0.616
			LDpred2-auto	1.59 (1.08-2.34)	0.019	0.019	0.021	0.604
			LDpred2-grid-sp	1.74 (1.16-2.62)	0.007	0.026	0.028	0.622
			PANPRS	1.19 (0.82-1.72)	0.353	0.002	0.003	0.573
			mvL	1.60 (1.12-2.29)	0.011	0.025	0.026	0.609
			mvL(BLD-X)	1.49 (0.99-2.22)	0.054	0.025	0.019	0.591
BD	SZ	Cases = 124 NAARs = 442	C+T	1.27 (1.03-1.56)	0.023	0.009	0.008	0.573
			Thresholding	1.18 (0.97-1.45)	0.104	0.005	0.004	0.547
			Lassosum	1.25 (1.00-1.55)	0.046	0.008	0.006	0.578
			LDpred2-auto	1.17 (0.96-1.44)	0.118	0.005	0.004	0.539
			LDpred2-grid-sp	1.27 (1.03-1.56)	0.024	0.011	0.008	0.568
			PANPRS	0.98 (0.80-1.19)	0.824	8×10^{-5}	7×10^{-5}	0.497
			mvL	1.47 (1.19-1.81)	3×10^{-4}	0.030	0.021	0.611
			mvL(BLD-X)	1.58 (1.27-1.96)	3×10^{-5}	0.041	0.028	0.631
	BD	Cases = 205 NAARs = 442	C+T	1.49 (1.25-1.78)	7×10^{-6}	0.032	0.023	0.614
			Thresholding	1.59 (1.33-1.90)	2×10^{-7}	0.044	0.031	0.623
			Lassosum	1.21 (1.01-1.43)	0.034	0.007	0.005	0.563
			LDpred2-auto	1.95 (1.63-2.33)	5×10^{-13}	0.094	0.060	0.682
			LDpred2-grid-sp	2.02 (1.68-2.42)	8×10^{-14}	0.097	0.062	0.686
			PANPRS	0.98 (0.83-1.16)	0.837	7×10^{-5}	5×10^{-5}	0.491
			mvL	2.06 (1.72-2.47)	7×10^{-15}	0.106	0.068	0.695
			mvL(BLD-X)	2.19 (1.82-2.63)	1×10^{-16}	0.123	0.077	0.713
	SAD	Cases = 35 NAARs = 442	C+T	1.65 (1.16-2.34)	0.005	0.017	0.025	0.633
			Thresholding	1.54 (1.09-2.15)	0.013	0.010	0.018	0.643
			Lassosum	1.60 (1.09-2.35)	0.015	0.014	0.021	0.645
			LDpred2-auto	1.81 (1.29-2.53)	6×10^{-4}	0.025	0.036	0.665
			LDpred2-grid-sp	2.21 (1.54-3.17)	2×10^{-5}	0.049	0.061	0.711
			PANPRS	0.74 (0.53-1.04)	0.086	0.005	0.009	0.414
			mvL	2.62 (1.83-3.74)	1×10^{-7}	0.080	0.092	0.759
			mvL(BLD-X)	2.51 (1.78-3.53)	1×10^{-7}	0.076	0.088	0.741

[a]: SS: summary statistics
[b]: Odds ratio of trait and 95% confidence interval for an increase of 1 standard deviation in PRS
[c]: Squared Pearson correlation ($\boldsymbol{R^2}$) and coefficient of determination on the liability scale ($\boldsymbol{R^2_{\text{liab.}}}$)[1] of PRS and diagnosis status. Population prevalence was set to 1% for SZ, 2% for BD and 0.3% for SAD[2]
[d]: Non-affected adult relatives
[e]: Clumping (window size=250 kb, r^2 and p selected by validation) and thresholding (p selected by validation)
[f]: Multivariate Lassosum
[g]: BLD-X: Baseline linkage disequilibrium model-cross trait
[h]: SZ: schizophrenia, BD: bipolar disorder, SAD: schizoaffective disorder

Table S4: Predictive performance of PRS on bipolar disorder with and without psychosis in the Eastern Quebec schizophrenia and bipolar disorder study

SS[a]	Trait	n	Method	OR (95% CI)[b]	P-value	R^{2c}	$R^{2c}_{\text{liab.}}$	AUC
SZ[h]	BD (with psychosis)	Cases = 93 NAARs[d] = 442	C+T[e]	0.92 (0.74-1.15)	0.473	0.001	0.001	0.489
			Thresholding	0.99 (0.80-1.23)	0.959	0.000	0.000	0.489
			Lassosum	0.88 (0.71-1.10)	0.266	0.003	0.002	0.496
			LDpred2-auto	1.31 (1.04-1.64)	0.022	0.009	0.009	0.569
			LDpred2-grid-sp	1.15 (0.90-1.46)	0.259	0.003	0.003	0.545
			PANPRS	0.96 (0.76-1.20)	0.695	3×10^{-4}	3×10^{-4}	0.487
			mvL[f]	1.40 (1.09-1.78)	0.008	0.020	0.015	0.609
			mvL(BLD-X[g])	1.45 (1.13-1.87)	0.004	0.026	0.019	0.618
	BD (without psychosis)	Cases = 112 NAARs = 442	C+T	1.62 (1.24-2.1)	4×10^{-4}	0.033	0.023	0.615
			Thresholding	1.42 (1.14-1.78)	0.002	0.023	0.017	0.593
			Lassosum	1.40 (1.10-1.78)	0.009	0.017	0.013	0.593
			LDpred2-auto	1.47 (1.17-1.85)	0.001	0.026	0.019	0.607
			LDpred2-grid-sp	1.62 (1.26-2.09)	2×10^{-4}	0.038	0.027	0.639
			PANPRS	1.19 (0.97-1.46)	0.087	0.005	0.004	0.550
			mvL	1.50 (1.20-1.87)	4×10^{-4}	0.027	0.022	0.625
			mvL(BLD-X)	1.52 (1.22-1.88)	2×10^{-4}	0.027	0.023	0.621
BD	BD (with psychosis)	Cases = 93 NAARs = 442	C+T	1.41 (1.11-1.79)	0.005	0.017	0.014	0.602
			Thresholding	1.59 (1.25-2.03)	2×10^{-4}	0.035	0.027	0.622
			Lassosum	1.03 (0.81-1.30)	0.815	1×10^{-4}	1×10^{-4}	0.522
			LDpred2-auto	2.03 (1.60-2.57)	6×10^{-9}	0.085	0.063	0.688
			LDpred2-grid-sp	2.00 (1.57-2.54)	2×10^{-8}	0.079	0.058	0.681
			PANPRS	1.00 (0.80-1.25)	0.986	0.000	0.000	0.496
			mvL	2.09 (1.65-2.66)	2×10^{-9}	0.090	0.067	0.699
			mvL(BLD-X)	2.23 (1.75-2.85)	8×10^{-11}	0.108	0.079	0.718
	BD (without psychosis)	Cases = 112 NAARs = 442	C+T	1.57 (1.27-1.93)	2×10^{-5}	0.028	0.025	0.623
			Thresholding	1.60 (1.29-1.98)	2×10^{-5}	0.031	0.027	0.625
			Lassosum	1.40 (1.14-1.74)	0.002	0.015	0.014	0.597
			LDpred2-auto	1.90 (1.52-2.38)	2×10^{-8}	0.064	0.050	0.676
			LDpred2-grid-sp	2.04 (1.62-2.55)	7×10^{-10}	0.071	0.057	0.690
			PANPRS	0.97 (0.78-1.20)	0.764	2×10^{-4}	2×10^{-4}	0.487
			mvL	2.03 (1.63-2.53)	2×10^{-10}	0.075	0.060	0.691
			mvL(BLD-X)	2.13 (1.71-2.66)	2×10^{-11}	0.085	0.067	0.709

[a]: SS: summary statistics
[b]: Odds ratio of trait and 95% confidence interval for an increase of 1 standard deviation in PRS
[c]: Squared Pearson correlation ($\boldsymbol{R^2}$) and coefficient of determination on the liability scale ($\boldsymbol{R^2}_{\text{liab.}}$)[1] for PRS and diagnosis status. Population prevalence was set to 0.9% for BD with psychosis and 1.1% for BD without psychosis (proportional to sample frequencies)
[d]: Non-affected adult relatives
[e]: Clumping (window size=250 kb, r^2 and p selected by validation) and thresholding (p selected by validation)
[f]: Multivariate Lassosum
[g]: BLD-X: Baseline linkage disequilibrium model-cross trait
[h]: SZ: schizophrenia, BD: bipolar disorder

References

[1] Lee, S. H., Goddard, M. E., Wray, N. R., and Visscher, P. M. (2012). A better coefficient of determination for genetic profile analysis. Genet Epidemiol 36, 214–24.

[2] Perälä, J., Suvisaari, J., Saarni, S. I., Kuoppasalmi, K., Isometsä, E., Pirkola, S., Partonen, T., Tuulio-Henriksson, A., Hintikka, J., Kieseppä, T. et al. (2007). Lifetime prevalence of psychotic and bipolar I disorders in a general population. Arch Gen Psychiatry 64, 19–28.
